# Transient receptor potential vanilloid 4 blockage attenuates pyroptosis in hippocampus of mice following pilocarpine‑induced status epilepticus

**DOI:** 10.1186/s40478-025-01990-5

**Published:** 2025-04-10

**Authors:** Lihan Liu, Yue Wang, Xiaolin Wang, Guowen Zhang, Sha Sha, Rong Zhou, Yimei Du, Chunfeng Wu, Lei Chen

**Affiliations:** 1 Department of Physiology, Nanjing Medical University, No. 101, Longmian Ave, Nanjing, Jiangsu Province 211166 P.R. China; 2 Department of Neurology, Children’s Hospital of Nanjing Medical University, No.8, Jiangdong South Road, Nanjing, Jiangsu Province 211166 P. R. China; 3 Research Center of Ion Channelopathy, Institute of Cardiology, Union Hospital, Tongji Medical College, Huazhong University of Science and Technology, Wuhan, Hubei Province 430022 P.R. China

**Keywords:** Temporal lobe epilepsy, Transient receptor potential vanilloid 4, Pyroptosis, Calpain, Caspase-1, Gasdermin D

## Abstract

Pyroptosis contributes to the neuronal damage that occurs during epilepsy. Calcium-activated neutral protease (calpain) dissociates cysteinyl aspartate specific proteinase-1 (caspase-1, cas-1) from the cytoskeleton, and the activated cas-1 is responsible for the production of N-terminus of gasdermin D (N-GSDMD), the final executor of pyroptosis. Blocking transient receptor potential vanilloid 4 (TRPV4) can reduce neuronal injury in temporal lobe epilepsy (TLE) model mice. This study investigated the role of TRPV4 in pyroptosis during TLE. In the hippocampus of pilocarpine-induced status epilepticus (PISE) mice, the ratio of inactive calpain 1 protein level to its total protein level (inactive/total calpain 1) significantly decreased, while the ratio of inactive calpain 2 protein level to its total protein level remained unchanged. The protein levels of NLRP3, cleaved cas-1 (c-cas-1), interleukin (IL)-1β, and N-GSDMD increased, with more GSDMD-immunofluorescence-positive (GSDMD^+^) cells and fewer surviving pyramidal neurons observed in the hippocampus of PISE mice. Calpain inhibition with MDL-28170 reversed these changes, except for the elevated NLRP3 levels. Inhibitors targeting NLRP3 (MCC950) and cas-1 (Ac-YVAD-cmk) blocked the increase in c-cas-1, IL-1β, and N-GSDMD levels in the hippocampus of PISE mice. TRPV4 inhibition via HC-067047 increased the inactive/total calpain 1 ratio, decreased NLRP3, c-cas-1, IL-1β, and N-GSDMD protein levels, reduced GSDMD^+^ cells number, and improved pyramidal neuron survival in the hippocampus of PISE mice. Conversely, TRPV4 activation with GSK1016790A decreased the inactive/total calpain 1 ratio, elevated NLRP3, c-cas-1, IL-1β, and N-GSDMD levels, and increased GSDMD^+^ cells number in the hippocampus. In the hippocampus of GSK1016790A-injected mice, the inactive/total calpain 1 ratio was increased by MDL-28170, and c-cas-1, IL-1β, and N-GSDMD protein levels were markedly attenuated by MDL-28170, MCC950, and Ac-YVAD-cmk, respectively. In conclusion, TRPV4 inhibition mitigates pyroptosis in PISE mice by downregulating the calpain 1–NLRP3/cas-1–GSDMD pathway, ultimately reducing neuronal damage.

**Supplementary Information:**

The online version contains supplementary material available at 10.1186/s40478-025-01990-5.

## Introduction

Epilepsy is a central nervous system disease caused by sudden abnormal discharges in brain neurons. Status epilepticus (SE) can cause irreversible brain damage. Research has found that, in rats with temporal lobe epilepsy (TLE), SE for 45 min can induce significant neuronal damage in the hippocampal CA3 region [1]. In pilocarpine-induced TLE mice, SE for 1 h reduced the numbers of surviving pyramidal neurons in the hippocampal CA1 and CA3 regions by 40–50% [2]. Neuronal damage impairs the synaptic structure, leading to the dysfunction of synaptic transmission and abnormal neuronal discharge. However, neuronal damage can stimulate neurogenesis in the dentate gyrus of the hippocampus. The over-proliferation of neural stem cells was found in TLE rodents, and more newborn neurons migrated to the portal region of the hippocampus, resulting in the formation of ectopic synaptic connections with the dentate gyrus [3]. Neuronal damage is an important change in the pathogenesis of TLE which plays an important role in promoting the progression of TLE, but the underlying mechanisms are not entirely clear.

Pyroptosis is a form of programmed cell death characterized by plasma membrane pore formation mediated by the gasdermin protein family [4]. In its canonical pathway, pyroptosis is triggered by inflammasomes such as the NOD-like receptor family pyrin domain containing 1 (NLRP1) or NOD-like receptor family pyrin domain containing 3 (NLRP3). These inflammasomes activate cysteine aspartic acid-specific protease-1 (caspase-1, cas-1), leading to the generation of its cleaved active form (c-cas-1) [5]. C-cas-1 then cleaves gasdermin D (GSDMD) to generate N-terminal fragments (N-GSDMD). These fragments oligomerize to form pores in the plasma membrane, causing osmotic cell swelling, release of pro-inflammatory cytokines (e.g., interleukin (IL)-1 beta (IL-1β), IL-18), and ultimately neuronal injury [5]. Critically, this pyroptotic cascade is directly associated with epilepsy pathogenesis. In both TLE patients and animal models, NLRP1 and cas-1 expression is significantly upregulated in the hippocampus, correlating with neuronal pyroptosis. Notably, knockdown of Nlrp1 or pharmacological inhibition of cas-1 not only attenuated neuronal damage but also reduced seizure frequency [6], suggesting pyroptosis as a therapeutic target in epilepsy.

Calcium-activated neutral protease (calpain) is a cysteine protease that is activated by an increase in intracellular calcium ion concentration ([Ca^2+^]_i_) [7]. Calpain is present in two forms, calpain 1 and calpain 2, which require micromolar and millimolar concentrations of calcium ion (Ca^2+^) for activation, respectively. Both calpain 1 and calpain 2 are widely expressed in the brain [7]. Calpain plays an important role in regulating the activation of cas-1. Activated calpain 1 can dissociate cas-1 from the cytoskeleton, and cas-1 then participates in inflammasome (e.g., NLRP3) assembly and activation [8]. Silencing calpain 1 alleviates myocardial ischemia-reperfusion injury in mice by inhibiting the NLRP3–cas-1 pathway [9]. The expression and activity of calpain 1 increase significantly during epilepsy, and the administration of a calpain inhibitor, such as MDL-28170, can significantly attenuate seizures [10]. However, whether calpain is responsible for the NLRP3 inflammasome and cas-1 activation and is related to pyroptosis during epilepsy has not yet been reported.

Transient receptor potential vanilloid 4 (TRPV4) is permeable to Ca^2+^, and activation of TRPV4 has been proven to increase [Ca^2+^]_i_ [11]. Bioinformatics analysis showed that *Trpv4* is a susceptibility gene for epilepsy, and TRPV4 is expressed in the glial cells and neurons in the brain. Activation of TRPV4 in astrocytes was found to promote neuroinflammation through the TRPV4–Ca^2+^–Yes-associated protein (YAP)–Signal transducer and activator of transcription 3 (STAT3) signaling pathway in mice with seizures, while TRPV4 inhibition in astrocytes attenuated neuroinflammation, reduced neuronal damage, and improved neurobehavioral function [12]. A study showed TRPV4 blockage reduced NLRP3 inflammasome expression and inflammatory factors levels in TLE mice [2]. These reports indicate that TRPV4 is involved in inflammation during epilepsy, but whether TRPV4 activation is responsible for activating calpain during epilepsy has not been reported.

The present study examined the effects of TRPV4 blockage on pyroptosis in mice following pilocarpine-induced status epilepticus (PISE) and further explored whether this action was related to the calpain 1–NLRP3/cas-1–GSDMD pathway.

## Materials and methods

### Animals

Male ICR mice, aged 6 weeks old and weighing 25–30 g, were used in this study. Mice were obtained from the Animal Core Facility of Nanjing University (Nanjing, China), where they were housed under controlled conditions (temperature, 23 °C ± 2 °C; relative humidity, 55% ± 5%; 12-h/12-h light/dark cycle) with free access to food and water. This study was approved by the ethics committee of Nanjing Medical University (No. IACUC2009007), and all animal experiments were performed in accordance with the Guidelines for Laboratory Animal Research set by Nanjing Medical University. Each experimental group contained nine mice (see Supplementary Methods and Supplementary Table 1).

### Preparation of PISE mice

Mice were first intraperitoneally (i.p.) injected with methylscopolamine (1 mg/kg), and 20 min later, injected (i.p.) with pilocarpine (300 mg/kg) to induce SE [2, 13]. Seizure behavioral severity was assessed using the Racine scale, which is defined as follows: category 1: immobility and facial twitching; category 2: head nodding; category 3: forelimb clonus; category 4: rearing; category 5: rearing followed by falling [2, 13, 14]. SE was defined as the onset of category 4–5 seizures lasting for 1 h that were terminated by injection (i.p.) of diazepam (10 mg/kg). If mice did not develop category 4–5 seizures within 30 min of pilocarpine injection, they were excluded from the study. Control mice were injected with the same volume of saline.

### Drug administration

TRPV4 agonist GSK1016790A, TRPV4 antagonist HC-067047, and cas-1 inhibitor Ac-YVAD-cmk were administered by intracerebroventricular (i.c.v.) injection [2, 13]. Mice were anesthetized with ketamine (100 mg/kg)/xylazine (10 mg/kg) via i.p. injection, and placed on a stereotaxic device (Kopf Instruments, Tujunga, CA, USA). A guide cannula of 23-gauge stainless steel tubing was implanted into the right lateral ventricle (0.3 mm posteriorly, 1.0 mm laterally, and 2.5 mm ventrally to the bregma) and anchored to the skull. Drugs were injected into the right lateral ventricle using a 26-gauge stainless steel needle (Plastics One, Roanoke, VA, USA) at the rate of 0.2 µL/min. The total injection volume was 2 µL. Calpain inhibitor MDL-28170 and NLRP3 inhibitor MCC950 were administered via i.p. injection [15, 16]. GSK1016790A (5 µM/2 µL/mouse) was administered daily for three consecutive days. HC-067047 (10 µM/2 µL/mouse) was first injected 1 h after SE was terminated and then once daily for 3 days. Ac-YVAD-cmk (200 ng/2 µL/mouse) was first injected 30 min before GSK1016790A or 1 h after pilocarpine injection, then once daily for 3 days. MDL-28170 (20 mg/kg) was injected 1, 5, and 9 h after GSK1016790A or pilocarpine injection, and then once daily for 3 days. MCC950 (20 mg/kg) was injected 30 min before and 6 h after GSK1016790A or pilocarpine injection, and then once daily for 3 days. The dosage of these drugs was selected according to previous studies [2, 13–20]. The control group mice were given the same volume of vehicle.

### Western blot analysis

Hippocampal samples were obtained 3 days after SE or 8 h after the last injection of drugs. Total protein was extracted using the whole cell lysis assay kit (cat. no. KGP250) from Nanjing KeyGen Biotech Co., Ltd. (Nanjing, China) according to the manufacturer’s protocols. The protein concentrations were determined using a BCA protein assay kit (cat. no. P0012, Beyotime Institute of Biotechnology, Shanghai, China). Equal amounts of protein (20 µg) were separated by sodium dodecyl sulfate-polyacrylamide gel electrophoresis and transferred to polyvinylidene difluoride membranes. The membranes were blocked using 5% non-fat milk in Tris-buffered saline (TBS)/Tween 20, and then incubated with primary antibodies against inactivated calpain 1 (cat. no. RP1-calpain-1, 1:1000), total calpain 1 (cat. no. RP3-calpain-1, 1:1000), inactivated calpain 2 (cat. no. RP2-calpain-2, 1:1000), and total calpain 2 (cat. no. RP3-calpain-2, 1:1000) (all from Triple Point Biologics, Forest Grove, OR, USA); NLRP3 (cat. no. bs-6655R, 1:500, Beijing Biosynthesis Biotechnology, Beijing, China); c-cas-1 (cat. no. 89332, 1:1000, Cell Signaling Technology, Danvers, MA, USA); GSDMD (cat. no. AF-4012, 1:1000, Affinity Biosciences, Melbourne, Australia); IL-1β (cat. no. 16806-1-AP, 1:1000, Proteintech Group Inc., Wuhan, China); and glyceraldehyde-3-phosphate dehydrogenase (GAPDH, cat. no. AP0063, 1:1,000; Bioworld Technology, Minneapolis, MN, USA) at 4 °C overnight. After washing with TBST, the membranes were incubated with a horseradish peroxidase-labeled secondary antibody and developed using an ECL detection kit (Amersham Biosciences, Piscataway, NJ, USA). Band intensities were quantified using a Tanon Digital Gel Imaging Analysis System (Tanon**-**5200; Shanghai Tanon Science & Technology, Shanghai, China) and analyzed using the ImageJ software program (U.S. National Institutes of Health, Bethesda, MD, USA).

### Histological examination

Mice were anesthetized and transcardially perfused with ice-cold phosphate-buffered saline, followed by 4% paraformaldehyde either 8 h after the final drug injection or 3 days after the onset of SE. The excised brains were fixed overnight at 4 °C and subsequently dehydrated in 15% and 30% sucrose solutions. Coronal Sect. (12 μm thick) were prepared at the dorsal hippocampal level using a frozen microtome. For immunofluorescence staining, sections were blocked with goat serum and incubated overnight at 4 °C with a primary antibody against GSDMD (cat. no. AF-4012, 1:100, Affinity Biosciences, Melbourne, Australia). This was followed by incubation with a fluorescent secondary antibody (Alexa Fluor 488, cat. no. A11034, 1:200, Invitrogen, Carlsbad, CA, USA) for 1.5 h at room temperature. The sections were then mounted using antifade medium containing DAPI. GSDMD immunopositive (GSDMD^+^) cells in the hippocampus were visualized using a confocal laser-scanning microscope (Leica, Heidelberg, Germany). GSDMD^+^ cells were counted in every 10th section, with 20 sections analyzed per group. The total number of GSDMD^+^ cells in the hippocampus was estimated by multiplying the counted cells by 10 [21]. For toluidine blue staining, brains were removed, placed in fixative overnight at 4 °C, and subsequently processed for paraffin embedding. Coronal Sect. (5 μm thick) were cut at the hippocampal level and stained with toluidine blue. Pyramidal cells in the hippocampal CA1 and CA2/3 regions were identified using a light microscope (Olympus DP70, Olympus Corporation, Tokyo, Japan). The surviving neurons were counted in six sections per mouse, and the neuronal density was expressed as previously reported [2, 13]. For double immunostaining, free-floating sections were incubated with primary antibodies against GSDMD (cat. no. AF-4012, 1:100, Affinity Biosciences, Melbourne, Australia), neuronal nuclei (NeuN, cat. no. 26975-1-AP, 1:500, Proteintech Group Inc, Wuhan, China), glial fibrillar acidic protein (GFAP, cat. no. MAB360, 1:500, Millipore, Massachusetts, USA), and ionized calcium-binding adapter molecule 1 (Iba-1, cat. no. Ab178847, 1:500, Abcam, Cambridge, UK). The sections were then incubated with either biotin-conjugated goat anti-mouse IgG (cat. no. ab6788, 1:2000) or biotin-conjugated rabbit anti-goat IgG (cat. no. ab6740, 1:100) (both from Abcam, Cambridge, UK). GSDMD^+^ cells co-expressing NeuN (GSDMD^+^/NeuN^+^), GFAP (GSDMD^+^/GFAP^+^), or Iba-1 (GSDMD^+^/Iba-1^+^) were visualized using a light microscope (Olympus DP70, Olympus Corporation, Tokyo, Japan).

### Chemicals

HC-067047 (cat. no. HY-100208), MCC950 (cat. no. HY-12815 A), and MDL-28170 (cat. no. HY-18236) were obtained from MedChem Express (Shanghai, China). While pilocarpine (cat. no. 14487), ethylscopolamine (cat. no. 23862), ketamine (cat. no. 11630), Ac-YVAD-cmk (cat. no. 10014), and xylazine (cat. no. 14113) were obtained from Cayman Chemical Company (Ann Arbor, MI, USA). Unless otherwise stated, all other chemicals were obtained from Sigma Chemical Company (St. Louis, MO, USA).

### Statistical analysis

Data were analyzed using the Statistical Package for the Social Sciences, version 18.0 (IBM Corp., Armonk, NY, USA). The normality of the distribution was assessed using the Shapiro–Wilk test, and variance homogeneity was assessed using Levene’s test before statistical analysis. When the data followed a normal distribution with homogeneous variance, independent-samples t-tests were performed for statistical analysis, and the results were expressed as mean ± standard deviation. In cases where the data did not follow a normal distribution or exhibited heterogeneous variance, non-parametric Mann–Whitney U tests were conducted, with the results presented as boxplots showing the median and interquartile range (25th–75th percentile). Significance levels were set at *P* < 0.05 (see Supplementary Table 2, Table 3 and Table 4). The protein levels in PISE mice were normalized to those of control mice. The protein levels in drug-treated PISE model mice were normalized to those of vehicle-treated PISE mice. The protein levels in drug-injected mice were normalized to those of vehicle-injected mice.

## Results

### Changes in calpain, NLRP3, cas-1, IL-1β, and GSDMD expression in the hippocampus following PISE

**Fig. 1:**
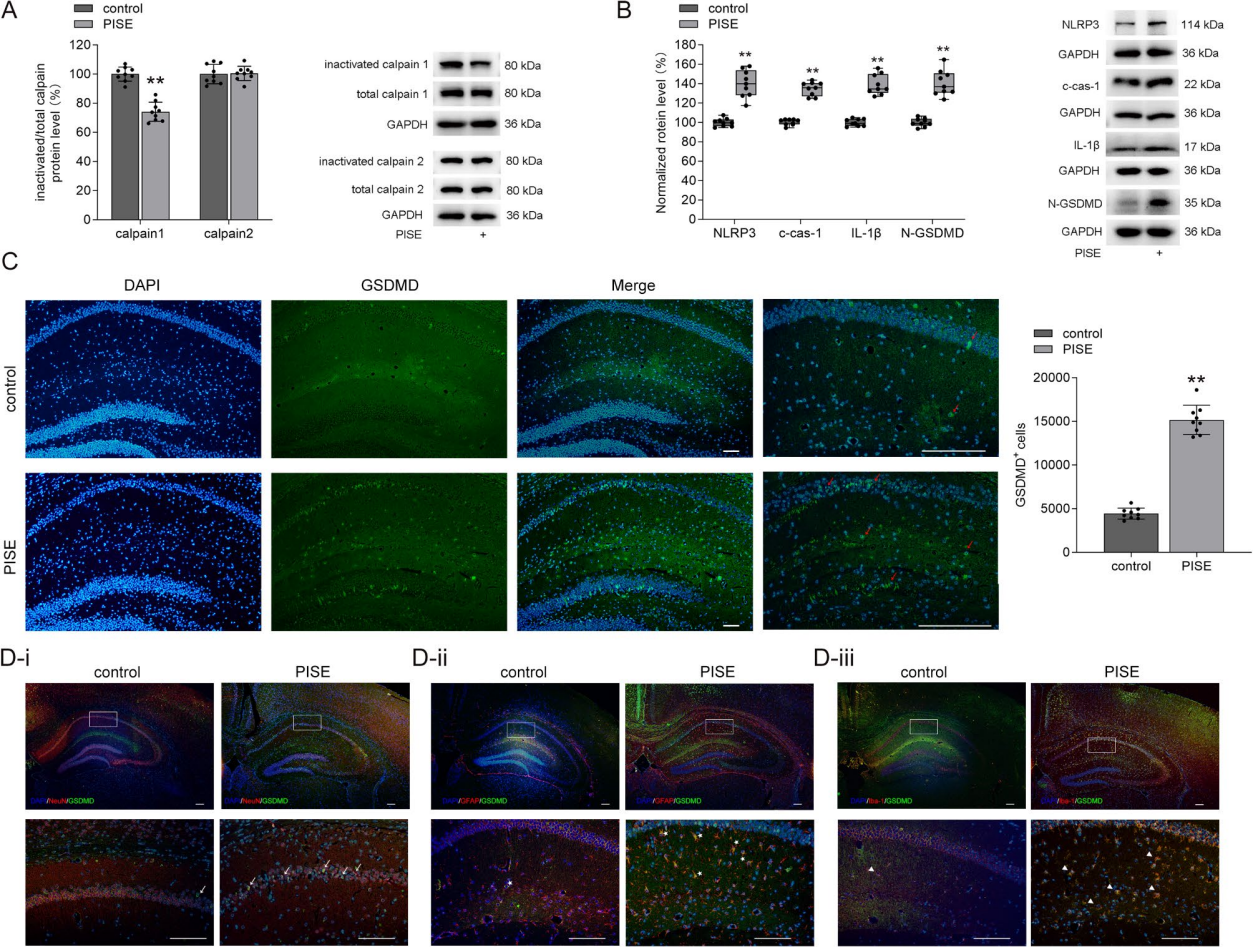
Changes in calpain 1–NLRP3/cas-1–GSDMD pathway in the hippocampus of PISE mice. **(A)** The inactive/total calpain 1 ratio markedly decreased (independent-samples *t* test, *t* = 9.42, *P* < 0.01) while the inactive/total calpain 2 ratio did not change (independent-samples *t* test, *t* = − 0.16, *P* = 0.87) in the hippocampus of PISE mice. **(B)** The protein levels of NLRP3 (Mann–Whitney U test, *P* < 0.01), c-cas-1 (Mann–Whitney U test, *P* < 0.01), IL-1β (Mann–Whitney U test, *P* < 0.01), and N-GSDMD (Mann–Whitney U test, *P* < 0.01) increased markedly in the hippocampus of PISE mice. **(C)** The number of GSDMD^+^ cells (red arrow) increased markedly in the hippocampus in PISE mice (independent-samples *t* test, *t* = − 18.01, *P* < 0.01). **(D)** GSDMD^+^/NeuN^+^ (D-i, white arrow), GSDMD^+^/GFAP^+^ (D-ii, white star) and GSDMD^+^/Iba-1^+^ (D-iii, white triangle) cells in the hippocampus of control and PISE mice. Scale = 50 μm. ×4 objective for the top row in D-i, D-ii and D-iii; ×10 objective for C-DAPI, C-GSDMD, C-Merge; ×20 objective for C-enlarge and the down row in D-i, D-ii and D-iii. **P < 0.01 vs. control

Divergences in calpain expression have been reported in epileptic rodent models [22, 23]. In this study, we first examined the protein levels of calpain 1 and calpain 2 in the hippocampus following PISE. As shown in Fig. 1A, the ratio of inactive calpain 1 protein level to its total protein level (inactive/total calpain 1) in the hippocampus of PISE mice was significantly lower than that of the control group, indicating increased calpain 1 activity. In contrast, the ratio of inactive calpain 2 protein level to its total protein level (inactive/total calpain 2) changed slightly in the hippocampus of PISE mice. These results suggest that calpain 1 activity increased following PISE, whereas calpain 2 activity remained largely unaffected. Previous studies have demonstrated that NLRP3 inflammasome expression is elevated during epilepsy [2]. Consistent with this, we observed a significant increase in NLRP3 protein levels in the hippocampi of PISE mice, accompanied by marked elevations in c-cas-1 and IL-1β protein levels (Fig. 1B). Notably, N-GSDMD protein levels were also elevated, and immunofluorescence staining revealed a significant increase in GSDMD^+^ cells in the hippocampus of PISE mice (Fig. 1C). Furthermore, the presence of GSDMD^+^/NeuN^+^ (Fig. 1D-i), GSDMD^+^/GFAP^+^ (Fig. 1D-i**i**), GSDMD^+^/Iba-1^+^ (Fig. 1D-i**ii**) cells confirmed that pyroptosis occurred in neurons, microglia, and astrocytes following PISE. These findings indicate the NLRP3 inflammasome activation and pyroptosis are key pathological changes following PISE.

### Effect of calpain inhibitor on calpain 1–NLRP3/cas-1–GSDMD signaling pathway following PISE

**Fig. 2:**
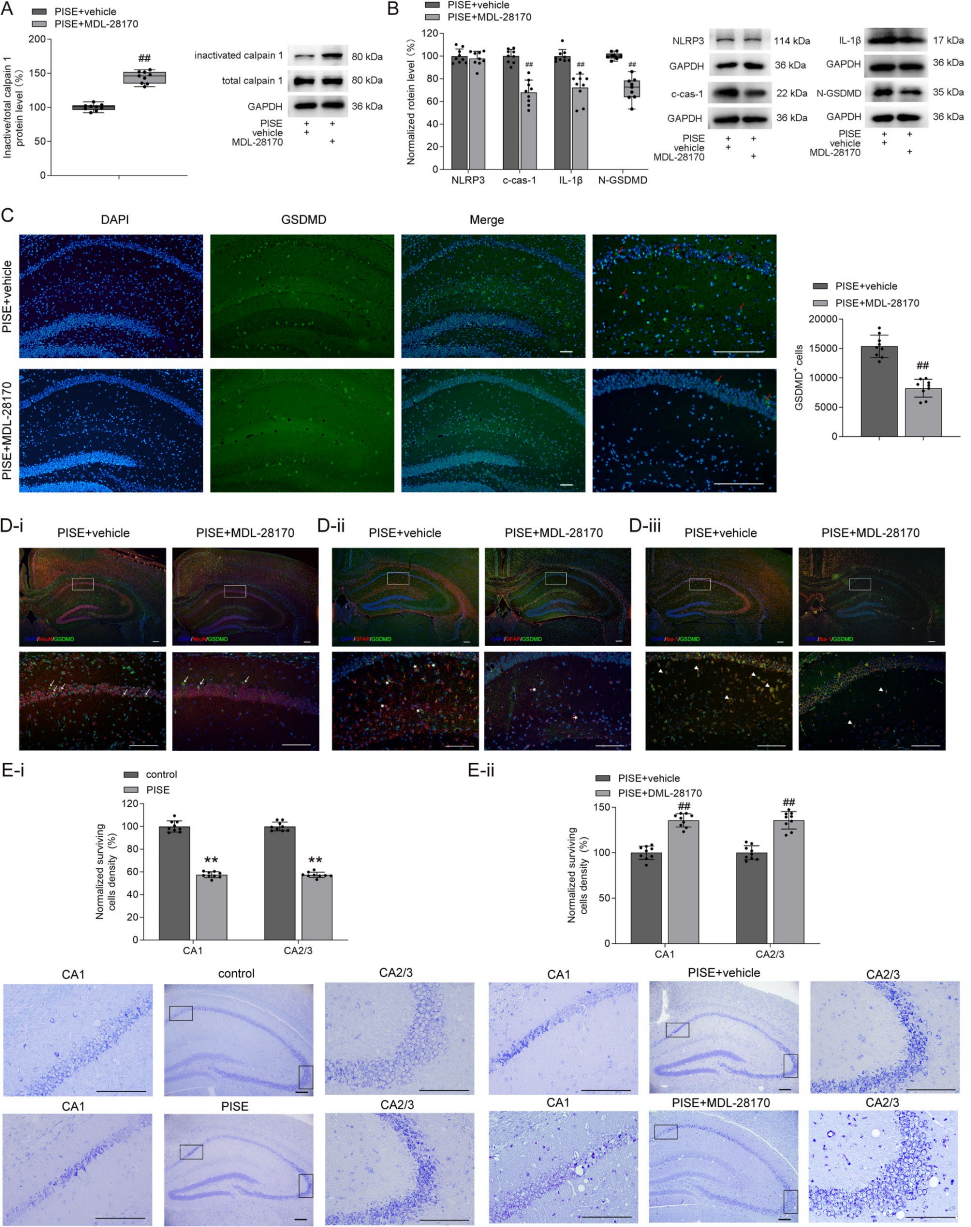
Effect of calpain inhibitor MDL-28170 on calpain 1–NLRP3/cas-1–GSDMD pathway in the hippocampus of PISE mice. **(A)** The calpain inhibitor MDL-28170 markedly increased the inactive/total calpain 1 rato in the hippocampus of PISE mice (Mann–Whitney U test, *P* < 0.01). **(B)** MDL-28170 did not affect NLRP3 protein levels in the hippocampus of PISE mice (independent-samples *t* test, *t* = 0.74, *P* = 0.47), but decreased c-cas-1 (independent-samples *t* test, *t* = 7.81, *P* < 0.01), IL-1β (independent-samples *t* test, *t* = 6.30, *P* < 0.01), and N-GSDMD protein levels (Mann–Whitney U test, *P* < 0.01). **(C)** MDL-28170 increased GSDMD^+^ cells (red arrow) number in the hippocampus of PISE mice (independent-samples *t* test, *t* = 8.83, *P* < 0.01). **(D)** GSDMD^+^/NeuN^+^ (D-i, white arrow), GSDMD^+^/GFAP^+^ (D-ii, white star) and GSDMD^+^/Iba-1^+^ (D-iii, white triangle) cells in the hippocampus of vehicle- and MDL-28179-treated PISE mice. **(E)** The numbers of surviving pyramidal neurons reduced in the hippocampal CA1 (independent-samples *t* test, *t* = 22.76, *P* < 0.01) and CA2/3 (independent-samples *t* test, *t* = 28.02, *P* < 0.01) area of PISE mice (E-i). Administration of MDL-28170 to PISE mice increased the numbers of surviving pyramidal cells in the hippocampal CA1 (independent-samples *t* test, *t* = 6.88, *P* < 0.01) and CA2/3 area (independent-samples *t* test, *t* = 6.15, *P* < 0.01) (E-ii). Scale = 50 μm. ×4 objective for the top row in D-i, D-ii and D-iii; ×10 objective for C-DAPI, C-GSDMD, C-Merge, and the middle column in E-i and E-ii; ×20 objective for C-enlarge, the down row in D-i, D-ii, and D-iii; ×40 objective for the left and right columns in E-i and E-ii. ^##^*P* < 0.01 vs. PISE + vehicle (ip.), **P < 0.01 vs. control

Calpain inhibition has been reported to alleviate epileptic seizure behaviors in PISE rats [22]. In our study, administration of the calpain inhibitor MDL-28170 had no effect on the inactive/total calpain 1 ratio in the hippocampus of normal mice (Supplementary Table 3) but significantly increased this ratio in PISE mice (Fig. 2A). These findings suggest that MDL-28170 effectively inhibits calpain 1 activation in the hippocampus following PISE. MDL-28170 administration did not alter the hippocampal protein levels of NLRP3, c-cas-1, IL-1β, or N-GSDMD in control mice (Supplementary Table 3). Treatment with MDL-28170 had no impact on NLRP3 protein levels, but significantly reduced hippocampal c-cas-1 protein levels in PISE mice (Fig. 2B). This reduction was accompanied by decreased IL-1β and N-GSDMD protein levels (Fig. 2B), along with a notable decline in the number of GSDMD^+^ cells (Fig. 2C), including GSDMD^+^/NeuN^+^ (Fig. 2D-i), GSDMD^+^/GFAP^+^ cells (Fig. 2D-i**i**) and GSDMD^+^/Iba-1^+^ (Fig. 2D-i**ii**). Importantly, MDL-28170 treatment significantly increased the number of surviving neurons in the hippocampus of PISE mice (Fig. 2E).

**Fig. 3:**
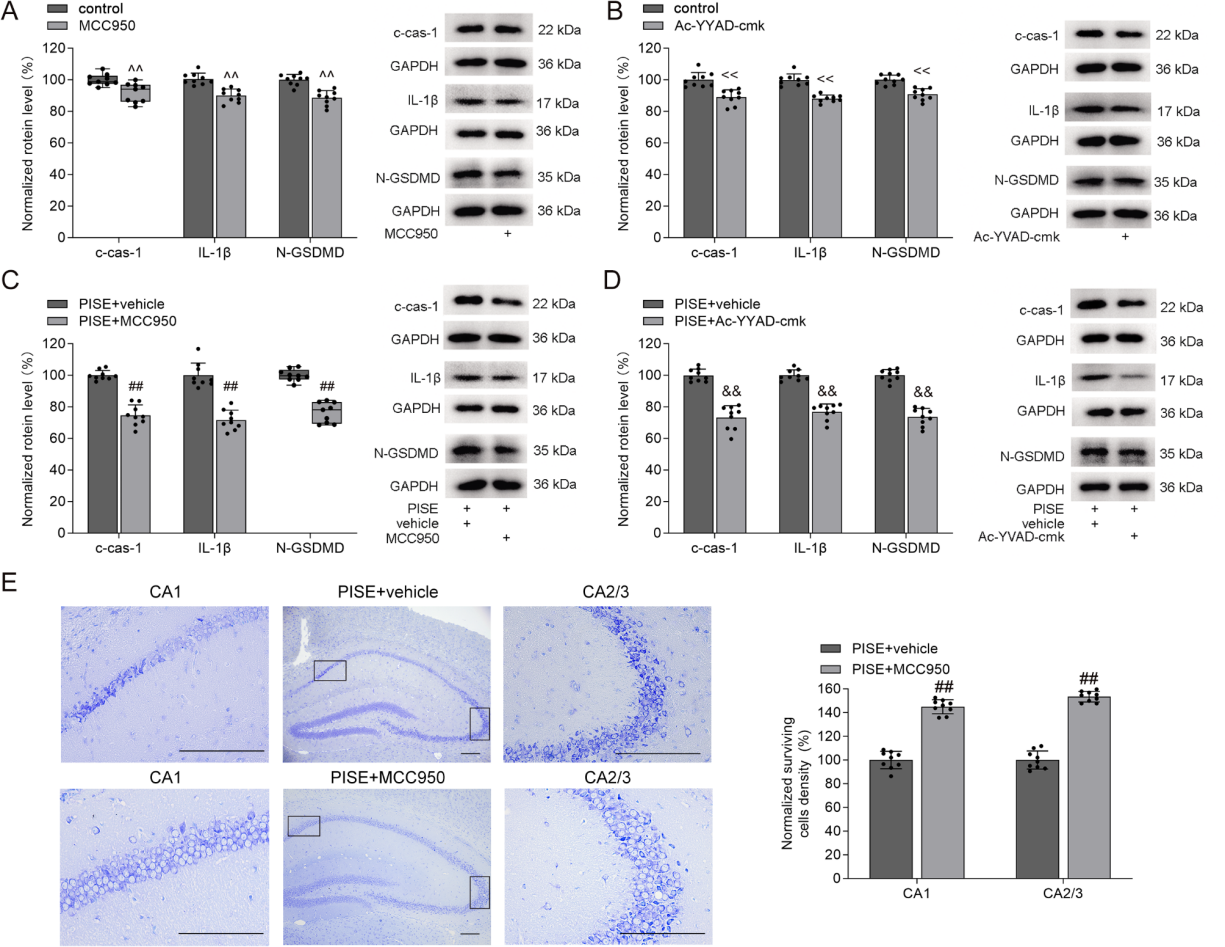
Effects of NLRP3 and cas-1 inhibitors on c-cas-1, IL-1β, and N-GSDMD protein levels in the hippocampus of PISE mice. **A** and **C**. NLRP3 inhibitor MCC950 decreased c-cas-1 (A, Mann–Whitney U test, *P* < 0.01; C, independent-samples *t* test, *t* = 10.47, *P* < 0.01), IL-1β (A, independent-samples *t* test, *t* = 5.75, *P* < 0.01; C, independent-samples *t* test, *t* = 8.50, *P* < 0.01), and N-GSDMD protein levels (A, independent-samples *t* test, *t* = 5.98, *P* < 0.01; C, Mann–Whitney U test, *P* < 0.01) in the hippocampus of control and PISE mice. **B** and **D**. Cas-1 inhibitor Ac-YVAD-cmk decreased c-cas-1 (B, independent-samples *t* test, *t* = 5.04, *P* < 0.01; D, independent-samples *t* test, *t* = 9.50, *P* < 0.01), IL-1β (B, independent-samples *t* test, *t* = 5.91, *P* < 0.01; D, independent-samples *t* test, *t* = 11.54, *P* < 0.01), and N-GSDMD protein levels (B, independent-samples *t* test, *t* = 8.14, *P* < 0.01; D, independent-samples *t* test, *t* = 12.08, *P* < 0.01) in the hippocampus of control and PISE mice. **E**. Administration of MCC950 to PISE mice increased the numbers of surviving pyramidal neurons in the hippocampal CA1 (independent-samples *t* test, *t* = 10.01, *P* < 0.01) and CA2/3 area (independent-samples *t* test, *t* = 14.50, *P* < 0.01). Scale = 50 μm. ×10 objective for the middle column in E; ×40 objective for the left and right columns in E. ^^^^*P* < 0.01 vs. control (ip.), ^<<^*P* < 0.01 vs. control (icv.), ^##^*P* < 0.01 vs. PISE + vehicle (ip.), ^&&^*P* < 0.01 vs. PISE + vehicle (icv.)

To further investigate the role of the NLRP3–cas-1 pathway, we examined the effects of MCC950, an NLRP3 inflammasome inhibitor, and Ac-YVAD-cmk, a cas-1 inhibitor. Administration of either MCC950 or Ac-YVAD-cmk reduced hippocampal c-cas-1, IL-1β, and N-GSDMD protein levels in control mice (Fig. 3A **and B**). In PISE mice, treatment with either MCC950 or Ac-YVAD-cmk markedly decreased hippocampal c-cas-1, IL-1β, and N-GSDMD protein levels compared to vehicle-treated PISE mice (Fig. 3C and D). Furthermore, MCC950-treated PISE mice displayed a significantly higher number of surviving neurons in the hippocampus compared to vehicle-treated PISE mice (Fig. 3E). These findings indicate that the NLRP3–cas-1–GSDMD pathway is activated following PISE. Collectively, our results demonstrate that inhibiting calpain 1 downregulates the calpain 1–NLRP3/cas-1–GSDMD signaling pathway, thereby suppressing pyroptosis and improving neuronal survival after PISE.

### Effects of TRPV4 antagonist on calpain 1–NLRP3/cas-1–GSDMD signaling pathway following PISE

**Fig. 4:**
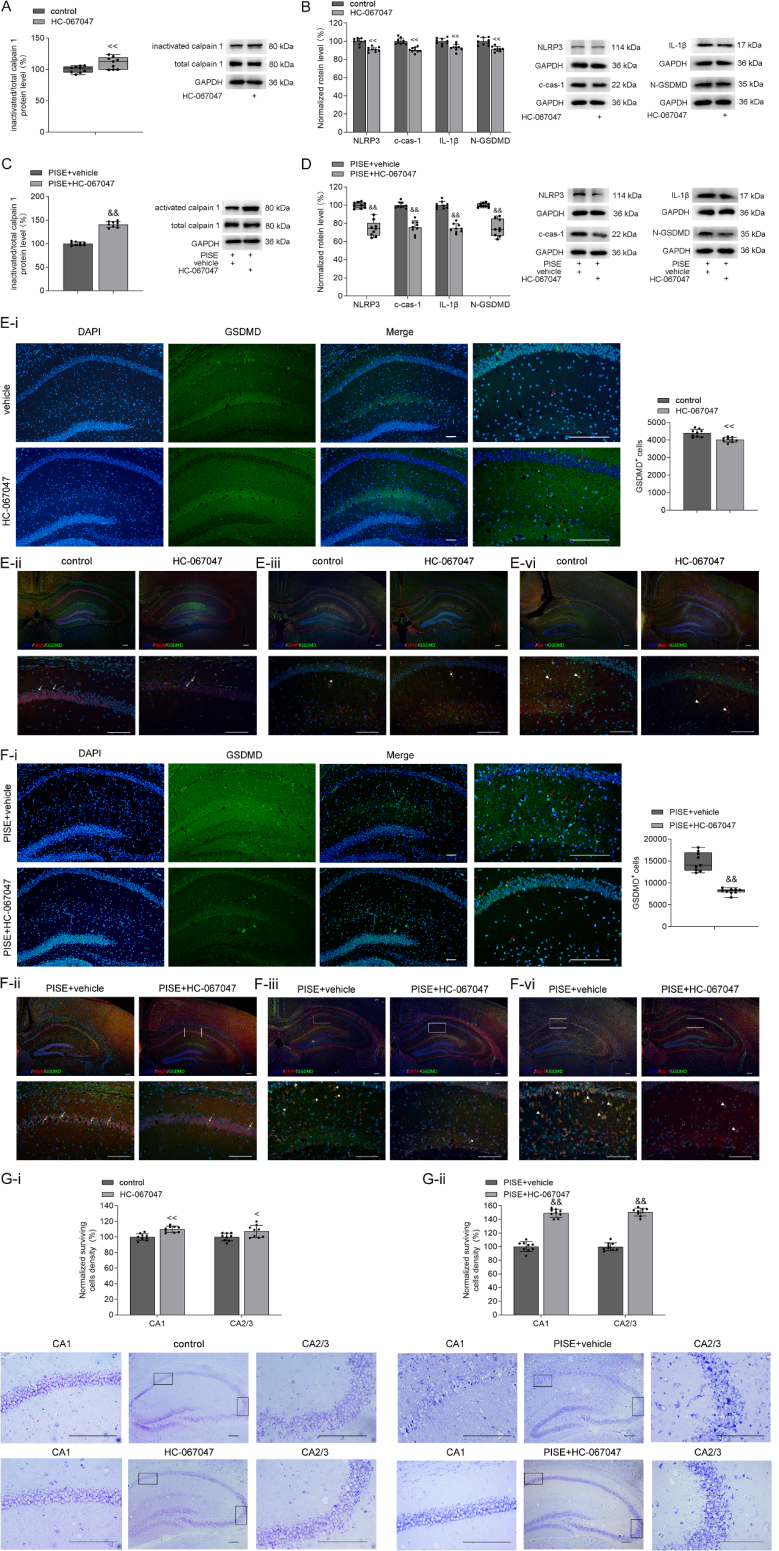
Effect of TRPV4 antagonist on calpain 1–NLRP3/cas-1–GSDMD pathway in the hippocampus of PISE mice. **A** and **C**. Administration of TRPV4 antagonist HC-067047 increased inactive/total calpain 1 ratio in the hippocampus of control (A, Mann–Whitney U test, *P* < 0.01) and PISE mice (C, independent-samples *t* test, *t* = − 17.09, *P* < 0.01). **B** and **D**. Administration of HC-067047 decreased NLRP3 (B, HC-067047: independent-samples *t* test, *t* = 6.49, *P* < 0.01; D, PISE + HC-067047: Mann–Whitney U test, *P* < 0.01), c-cas-1 (B, HC-067047: independent-samples *t* test, *t* = 6.53, *P* < 0.01; D, PISE + HC-067047: independent-samples *t* test, *t* = 9.38, *P* < 0.01), IL-1β (B, HC-067047: independent-samples *t* test, *t* = 4.23, *P* < 0.01; D, PISE + HC-067047: independent-samples *t* test, *t* = 12.31, *P* < 0.01), and N-GSDMD protein levels (B, HC-067047: independent-samples *t* test, *t* = 5.28, *P* < 0.01; D, PISE + HC-067047: Mann–Whitney U test, *P* < 0.01). **E** and **F**. Administration of HC-067047 decreased GSDMD^+^ cells (red arrow) numbers in the hippocampus of control (E-i, HC-067047: independent-samples *t* test, *t* = 4.34, *P* < 0.01) and PISE mice (F-i, PISE + HC-067047: Mann–Whitney U test, *P* < 0.01; white star). GSDMD^+^/NeuN^+^ (white arrow), GSDMD^+^/GFAP^+^ (white star) and GSDMD^+^/Iba-1^+^ (white triangle) cells in the hippocampus in control (E-ii, E-iii and E-iv) and PISE mice (F-ii, F-iii and F-iv) treated with either vehicle or HC-067047. **G.** HC-067047 treatment increased the numbers of surviving neurons in the hippocampal CA1 (G-i, independent-samples *t* test, *t* = 5.14, *P* < 0.01; G-ii, independent-samples *t* test, *t* = 10.12, *P* < 0.01) and CA2/3 (G-i, independent-samples *t* test, *t* = 2.35, *P* = 0.03; G-ii, independent-samples *t* test, *t* = 14.20, *P* < 0.01) area of control (G-i) and PISE mice (G-ii). Scale = 50 μm. ×4 objective for the top row in E-ii, E-iii, E-vi, F-ii, F-iii, F-vi; ×10 objective for E-i-DAPI, E-i-GSDMD, E-i-Merge, F-i-DAPI, F-i-GSDMD, F-i-Merge, and the middle columns in G-i and G-ii; ×20 objective for E-i-enlarge, F-i-enlarge, the down row in E-ii, E-iii, E-vi, F-ii, F-iii and F-vi; ×40 objective for the left and right columns in G-i, and G-ii. ^<^*P* < 0.05, ^<<^*P* < 0.01 vs. control (icv.), ^&&^*P* < 0.01 vs. PISE + vehicle (icv.)

Previous studies have reported that blocking TRPV4 can alleviate neuronal injury following SE [2, 13]. This study examined whether this protective effect was associated with the calpain 1–NLRP3/cas-1–GSDMD signaling pathway. Our results showed that treatment with the TRPV4 antagonist HC-067047 significantly increased the inactive/total calpain 1 ratio in the hippocampus of PISE mice (Fig. 4A), suggesting that TRPV4 is involved in calpain 1 activation following PISE. Furthermore, HC-067047 administration reduced hippocampal levels of NLRP3, c-cas-1, and IL-1β proteins in PISE mice (Fig. 4B and D), aligning with previous findings [2]. Notably, HC-067047 treatment significantly decreased N-GSDMD protein levels (Fig. 4D) and reduced the number of GSDMD^+^ cells in the hippocampus of PISE mice, including GSDMD^+^/NeuN^+^, GSDMD^+^/Iba-1^+^, and GSDMD^+^/GFAP^+^ cells (Fig. 4E and F). In addition, HC-067047 treatment improved neuronal survival in the hippocampus of PISE mice (Fig. 4G). These findings suggest that blocking TRPV4 may suppress the calpain 1–NLRP3/cas-1–GSDMD pathway, thereby inhibiting pyroptosis and reducing neuronal injury following PISE.

### Effects of TRPV4 agonist on calpain 1–NLRP3/cas-1–GSDMD pathway

**Fig. 5:**
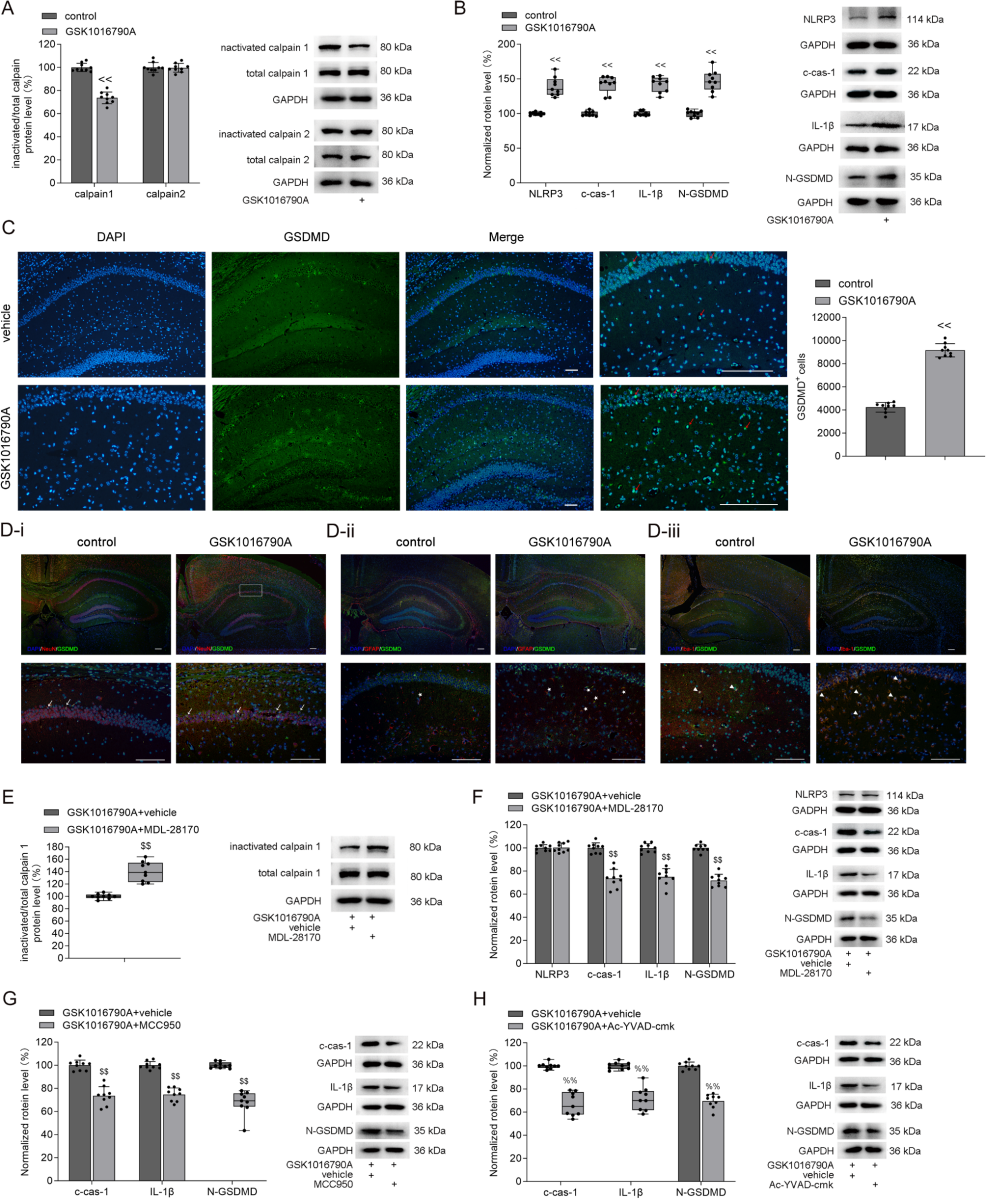
Effect of TRPV4 agonist on calpain 1-NLRP3/cas-1-GSDMD pathway in the hippocampus. **A**. The TRPV4 agonist GSK1016790A decreased inactive/total calpain 1 ratio (independent-samples *t* test, *t* = 12.72, *P* < 0.01) but did not change inactive/total calpain 2 ratio (independent-samples *t* test, *t* = 0.19, *P* = 0.86) in the hippocampus. **B** and **C**. GSK1016790A increased NLRP3 (B, Mann–Whitney U test, *P* < 0.01), c-cas-1 (B, Mann–Whitney U test, *P* < 0.01), IL-1β (B, Mann–Whitney U test, *P* < 0.01), and N-GSDMD (B, Mann–Whitney U test, *P* < 0.01) protein levels, and GSDMD^+^ cells (red arrow) number (C, independent-samples *t* test, *t* = − 20.96, *P* < 0.01) in the hippocampus. **D**. GSDMD^+^/NeuN^+^ (D-i, white arrow), GSDMD^+^/GFAP^+^ (D-ii, white star) and GSDMD^+^/Iba-1^+^ (D-iii, white triangle) cells in the hippocampus of control and GSK1016790A-injected mice. **E** and **F**. The calpain inhibitor MDL-28170 increased inactive/total calpain 1 ratio (E, Mann–Whitney U test, *P* < 0.01) and decreased c-cas-1 (F, independent-samples *t* test, *t* = 8.73, *P* < 0.01), IL-1β (F, independent-samples *t* test, *t* = 9.35, *P* < 0.01), and N-GSDMD protein levels (F, independent-samples *t* test, *t* = 13.52, *P* < 0.01) but did not affect NLRP3 protein levels (F, independent-samples *t* test, *t* = − 0.20, *P* = 0.85) in the hippocampus of GSK1016790A-injected mice. **G** and **H**. The NLRP3 inhibitor MCC950 (G) and cas-1 inhibitor Ac-YVAD-cmk (H) decreased c-cas-1 (G, independent-samples *t* test, *t* = 8.17, *P* < 0.01; H, Mann–Whitney U test, *P* < 0.01), IL-1β (G, independent-samples *t* test, *t* = 11.62, *P* < 0.01; H, Mann–Whitney U test, *P* < 0.01) and N-GSDMD protein levels (G, Mann–Whitney U test, *P* < 0.01; H, independent-samples *t* test, *t* = 13.38, *P* < 0.01) in the hippocampus in GSK1016790A-injected mice. Scale = 50 μm. ×4 objective for the top row in D-i, D-ii and D-iii; ×10 objective for C-DAPI, C-GSDMD and C-Merge; ×20 objective for C-enlarge, the down row in D-i, D-ii and D-iii. ^<<^*P* < 0.01 vs. Control (icv.), ^$$^*P* < 0.01 vs. GSK1016790A + vehicle, ^%%^*P* < 0.01 vs. GSK1016790A + vehicle

We also investigated the effect of TRPV4 activation on the calpain 1–NLRP3/cas-1–GSDMD pathway. As shown in Fig. 5A, the ratio of inactive/total calpain 1 significantly decreased in GSK1016790A-injected mice, whereas the ratio of inactive/total calpain 2 remained unchanged. These results suggest that TRPV4 activation selectively enhanced calpain 1 activity. In addition, hippocampal levels of NLRP3, c-cas-1, and IL-1β proteins were markedly elevated in GSK1016790A-injected mice, accompanied by an increase in N-GSDMD protein levels (Fig. 5B). A higher number of GSDMD^+^ cells, including GSDMD^+^/NeuN^+^, GSDMD^+^/Iba-1^+^, and GSDMD^+^/GFAP^+^ cells, were also observed in the hippocampus of these mice (Fig. 5C and D). To further explore the role of calpain 1, MDL-28170 was administered to GSK1016790A-injected mice. This treatment significantly increased the inactive/total calpain 1 ratio in the hippocampus (Fig. 5E). Moreover, MDL-28170 administration markedly reduced the hippocampal protein levels of c-cas-1, IL-1β, and N-GSDMD in GSK1016790A-injected mice (Fig. 5F). MDL-28170 treatment had no effect on NLRP3 protein levels in the hippocampus of GSK1016790A-injected mice (Fig. 5F). Finally, administration of either MCC950 (Fig. 5G) or Ac-YVAD-cmk (Fig. 5H) significantly reduced the hippocampal protein levels of c-cas-1, IL-1β, and N-GSDMD in GSK1016790A-injected mice. These findings demonstrate that TRPV4 activation enhances calpain 1–NLRP3/cas-1–GSDMD pathway, ultimately promoting pyroptosis.

## Discussion

SE is known to cause irreversible brain damage, with substantial loss of hippocampal pyramidal neurons observed in patients who succumb to SE [24]. In kainic acid-induced TLE rats, hippocampal neuronal death becomes evident 3 days after SE and peaked 6 days after SE [25]. Similarly, our previous study demonstrated that SE resulted in approximately 40–50% neuronal death in the hippocampus 3 days following PISE in mice [2, 13]. While neuronal damage is a significant pathological hallmark of epilepsy, the underlying mechanisms are complex and yet to be fully elucidated.

Elevated inflammatory cytokine levels are consistently detected in surgically resected brain tissue from TLE patients and TLE rodent models prepared with epileptogenic drugs or electrical stimulation [6, 26]. The inflammasome is a multiprotein complex comprising an inflammasome sensor molecule, the adaptor protein ASC (apoptosis-associated speck-like protein containing a caspase recruitment domain), and cas-1 [4]. Upon activation, the inflammasome sensor molecule oligomerizes and recruits ASC, which is essential for cas-1 activation and the subsequent production of c-cas-1 [27, 28]. C-cas-1 then cleaves pro-IL-1β and GSDMD, generating mature IL-1β and N-GSDMD, respectively. N-GSDMD forms membrane pores, triggering pyroptosis in neurons and glial cells [29, 30]. In the present study, increased N-GSDMD protein levels and a greater number of GSDMD^+^ cells were observed in the hippocampi of PISE mice (Fig. 1). Furthermore, GSDMD^+^/NeuN^+^, GSDMD^+^/Iba-1^+^, and GSDMD^+^/GFAP^+^ cells were identified in the hippocampi of PISE mice (Fig. 1), indicating that pyroptosis occurs in neurons, microglia, and astrocytes following PISE. Pyroptosis in neurons may directly contribute to neuronal death, whereas pyroptosis in glial cells can promote the release of inflammatory factors, amplifying the inflammatory response and further exacerbating neuronal damage [28, 29].

Cas-1 is a crucial enzyme responsible for generating N-GSDMD. In pentylenetetrazol-induced TLE rats and patients with drug-resistant TLE, hippocampal cas-1 expression is significantly elevated and positively correlates with the severity of neuronal damage. Inhibiting cas-1 has been shown to reduce neuronal loss, decrease seizure incidence, and delay seizure onset [6]. The NLRP3 inflammasome is one of the most important inflammasomes involved in cas-1 activation [27, 28]. In our study, both NLRP3 inflammasome and cas-1 inhibitors markedly reduced c-cas-1, IL-1β, and N-GSDMD protein levels following PISE (Fig. 3), suggesting that the NLRP3–cas-1–GSDMD pathway is activated during TLE. Calpain, a well-known cytosolic cysteine protease, requires intracellular Ca²⁺ for its enzymatic activity. Calpains are involved in various cellular processes, including cytoskeletal remodeling, cell cycle regulation, signal transduction, cell differentiation, apoptosis, and pyroptosis [31]. Importantly, activated calpain facilitates the release of cas-1 from its sequestration in the cytoskeleton, a crucial step for NLRP3 inflammasome assembly and activation [8]. Increased calpain expression has been reported in resected brain tissues from patients with epilepsy [32]. Similarly, pilocarpine-induced TLE rats exhibit elevated calpain activity and expression, which correlates with neurodegeneration and seizure occurrence [10]. In our study, calpain 1 activity selectively increased following PISE (Fig. 1), and this elevation was effectively suppressed by the calpain inhibitor MDL-28170 (Fig. 2). NLRP3 transcription is primarily regulated by the NF-κB signaling pathway [28]. Calpain 1 has a bidirectional regulatory effect on NF-κB activation. On one hand, calpain 1 enhances NF-κB signaling by promoting IκB degradation. On the other hand, calpain 1 can cleave the p65 subunit of NF-κB, resulting in its inactivation [33, 34]. In our study, MDL-28170 administration did not alter NLRP3 protein levels in PISE mice (Fig. 2), suggesting that MDL-28170 may stabilize the NF-κB inhibitory complex by preventing IκBα degradation. This stabilization could counteract the NF-κB-mediated upregulation of NLRP3 transcription, ultimately resulting in no significant change in NLRP3 protein levels. In this study, MDL-28170 administration significantly reduced c-cas-1 levels (Fig. 2), indicating that calpain 1 primarily regulates the assembly and activation of the NLRP3 inflammasome rather than its expression. NLRP3 inflammasome is predominantly expressed in glial cells (microglia and astrocytes) [28]. Here, administration of MCC950, an NLRP3 inflammasome inhibitor, significantly increased hippocampal neuronal survival in PISE mice (Fig. 3), indicating the role of NLRP3 inflammasome activation in glial cells in promoting neuronal damage. Moreover, inhibiting calpain 1 significantly reduced IL-1β and N-GSDMD protein levels, decreased the number of GSDMD^+^ cells, and increased hippocampal neuronal survival in PISE mice (Fig. 2). These findings suggest that the hyperactivation of the calpain 1–NLRP3–cas-1–GSDMD pathway plays a pivotal role in promoting pyroptosis and neuronal injury following PISE. However, the precise mechanism underlying calpain 1 activation during TLE remains to be elucidated.

Recent studies have highlighted the involvement of TRPV4 in the pathogenesis of epilepsy [2, 11, 13]. Blocking TRPV4 has been shown to mitigate hippocampal neuronal damage in epileptic mice by inhibiting glial cells activation and reducing the inflammatory response [2]. There is evidence that TRPV4 activation is related to pyroptosis. For instance, administration of a TRPV4 antagonist or TRPV4 knockdown effectively prevented pyroptosis in the hippocampus of lipopolysaccharide (LPS)-treated mice [35]. Additionally, TRPV4 inhibition suppressed inflammatory pyroptosis and preserved the integrity of the colonic tight junction barrier, suggesting a potential therapeutic strategy for colonic injury in endotoxemia [36]. In chronic obstructive pulmonary disease (COPD), TRPV4 was found to be upregulated in airway epithelial cells from both patients and cigarette smoke-exposed mouse models. This upregulation activates the NLRP3–cas-1–GSDMD cascade, inducing pyroptosis and contributing to disease progression [37]. In the present study, treatment with a TRPV4 antagonist significantly reduced N-GSDMD protein levels and the number of GSDMD^+^ cells in the hippocampus of PISE mice (Fig. 4). Conversely, TRPV4 activation increased N-GSDMD protein levels and the number of GSDMD^+^ cells, including GSDMD^+^/NeuN^+^, GSDMD^+^/Iba-1^+^, and GSDMD^+^/GFAP^+^ cells in the hippocampus (Fig. 5). These findings demonstrate that TRPV4 plays a crucial role in mediating pyroptosis in the hippocampus following PISE.

Calpain activation is a well-established mediator of inflammasome regulation and pyroptotic signaling [21, 31]. Previous studies have demonstrated that transient receptor potential vanilloid (TRPV) family channels, including TRPV1 and TRPV6, modulate calpain activity via Ca²⁺ influx in diverse pathological contexts. For instance, TRPV1 activation enhances calpain 1 activity in macrophage foam cells by mediating [Ca²⁺]_i_ elevation [38], and TRPV1–Ca^2+^–calpain signaling plays a dominant role in the capsaicin-induced ablation of nociceptive terminals [39]. TRPV6-driven [Ca²⁺]_i_ elevation induces apoptosis in lung cancer cells through calpain 1/2 activation [40]. Notably, TRPV4, another member of the TRPV subfamily, shares similar effect with these channels in regulating intracellular Ca²⁺ dynamics. TRPV4 activation elevates [Ca²⁺]_i_ either directly by mediating Ca^2+^ influx or indirectly via NMDA receptor potentiation [41, 42], making it a plausible upstream regulator of calpain in neuroinflammatory cascades. Given calpain’s involvement in inflammasome regulation and pyroptosis, along with TRPV4’s established role in calcium signaling and neuroinflammation, we hypothesized that TRPV4 activation could influence the calpain-mediated pathway in epilepsy. Our study supports this hypothesis, as pharmacological inhibition of TRPV4 significantly reduced calpain 1 activity in the hippocampus following PISE (Fig. 4). Conversely, TRPV4 activation markedly increased calpain 1 activity, which was reversed by the calpain inhibitor MDL-28170 (Fig. 5). These findings indicate that TRPV4-mediated [Ca²⁺]i elevation is a key driver of calpain 1 activation in TLE. Furthermore, TRPV4 may promote pyroptosis through a multifaceted mechanism. TRPV4-induced [Ca²⁺]i elevation activates calpain 1, which facilitates the release of cas-1 from cytoskeletal sequestration. Simultaneously, TRPV4 enhances NF-κB signaling [2], leading to increased NLRP3 expression and promoting inflammasome assembly. This cascade culminates in NLRP3 inflammasome activation, generating c-cas-1 and N-GSDMD, ultimately driving pyroptotic cell death. Notably, inhibition of calpain reversed TRPV4-induced increases in c-cas-1, IL-1β, and N-GSDMD levels, confirming that calpain functions as a critical downstream effector of TRPV4 in pyroptosis (Fig. 5). Taken together, our findings establish TRPV4 as a pivotal regulator of calpain-1-NLRP3-driven pyroptosis in TLE, linking calcium dyshomeostasis to neuroinflammation and neuronal injury. Whether additional mechanisms are involved in TRPV4-mediated neuroinflammation and pyroptosis remains to be further investigated.

NLRP3 inflammasome activation stimulates the production of inflammatory cytokines, which can further enhance its activation. Moreover, pyroptosis leads to the release of inflammatory mediators and other cellular contents that serve as danger signals, promoting additional NLRP3 inflammasome activation and creating a positive feedback loop. Previous studies have shown that IL-1β treatment significantly increased calpain 1 activity in rat brain microvascular endothelial cells [43]. Similarly, tumor necrosis factor-alpha (TNF-α) treatment in human brain microvascular endothelial cells markedly elevated TRPV4 mRNA and protein levels [44]. Furthermore, TNF-α-pretreated endothelial cells exhibited an enhanced calcium response to the TRPV4 agonist GSK1016790A, indicating that TNF-α not only upregulates TRPV4 expression but also enhances its channel activity [45]. Activation of NLRP3 inflammasome can induce TNF-α release [28]. Consequently, it is proposed that activation of the NLRP3–cas-1–GSDMD pathway may further enhance calpain 1 or TRPV4 activity or expression, thereby reinforcing the positive feedback loop. However, it is important to note that these findings were observed in endothelial cells rather than neurons or glial cells. Further research is required to determine whether this mechanism also applies to neuronal or glial cells.

This study has several limitations. Frist, although specific antagonists and agonists for TRPV4, calpain, NLRP3, and cas-1 were used, potential off-target effects cannot be excluded. Future studies could employ alternative agonists/antagonists, knockout models, or high-throughput kinase profiling to validate target specificity. Second, male ICR mice were used to reduce hormonal variability associated with the estrous cycle [46]. More studies should assess sex-specific responses using gonadectomy and hormone supplementation. Third, this study focused on the acute phase of TLE, showing that TRPV4 blockade inhibited calpain 1–NLRP3-mediated pyroptosis on day 3 following PISE. Long-term evaluations of TRPV4 blockade on seizure behavior and cognitive outcomes in the chronic phase of TLE are warranted. Fourth, previous studies have reported increased expression of the NLRP1 and AIM2 inflammasomes following SE [47]. However, it remains to be investigated whether TRPV4 blockade influences the activity of these inflammasomes.

## Conclusion

This study reveals the pivotal role of the TRPV4–calpain 1–NLRP3–cas-1–GSDMD signaling axis in driving pyroptosis and hippocampal neuronal injury in epilepsy. TRPV4 acts as an upstream calcium channel that selectively activates calpain 1, initiating NLRP3 inflammasome assembly and activation. This cascade subsequently promotes cas-1-mediated GSDMD cleavage, generating N-GSDMD, which executes pyroptotic cell death. Notably, the identification of this “TRPV4–calpain 1–NLRP3–pyroptosis” axis in epilepsy distinguishes it from previously reported calpain–NLRP3 interactions seen in ischemia and metabolic disorders. Pharmacological inhibition of this pathway effectively reduces hippocampal neuronal damage, highlighting its potential as a therapeutic target for mitigating epilepsy-related neurotoxicity. While the precise mechanism by which TRPV4 activates calpain 1 remains to be fully elucidated, this study is the first to identify the TRPV4–calpain 1–NLRP3 axis as a critical driver of neuroinflammatory injury in epilepsy. This study provides a theoretical basis for developing targeted therapies to mitigate neuronal damage in epilepsy.

## Electronic supplementary material

Below is the link to the electronic supplementary material.

Supplementary Material 1

## Data Availability

Data is provided within the manuscript or supplementary information files.
