# Supplementary material for: Transient receptor potential vanilloid 4 blockage attenuates pyroptosis in hippocampus of mice following pilocarpine‑induced status epilepticus
Source: Acta Neuropathol Commun. 2025 Apr 10;13:73. doi: 10.1186/s40478-025-01990-5 (PMC11983898; doi:10.1186/s40478-025-01990-5)

Supplementary Methods

1. Preparation of PISE mice

Thirty-six mice were injected with methylscopolamine and pilocarpine, 27 of which displayed category 4–5 seizures. The PISE mice were randomly divided into a western blot analysis group and a histological examination for GSDMD staining and toluidine blue staining group. No PISE mice died during the experiment, and each PISE group contained 9 mice. Twenty-seven mice injected with methylscopolamine and saline were assigned to the control group. Control mice were randomly divided into a western blot analysis group and a histological examination for GSDMD staining and toluidine blue staining group, and each group contained 9 mice. None of the control mice died during the experiment.

2. Preparation of drug-treated mice

Ninety mice with implanted stainless steel tubes were randomly divided into four groups and injected (i.c.v.) with vehicle (control mice), HC-067047 (HC-067047-injected mice), GSK1016790A (GSK1016790A-injected mice), or Ac-YVAD-cmk (Ac-YVAD-cmk-injected mice). Mice injected with vehicle, HC-067047, or GSK1016790A were randomly divided into a western blot analysis group and a histological examination group. No mice died during the experiment, and each group contained 9 mice.

Fifty-four mice were randomly divided into three groups and injected (i.p.) with vehicle (control mice), MDL-28170 (MDL-28170-injected mice) or MCC950 (MCC950-injected mice). Mice in control and MDL-28170-injected group were

randomly divided into a western blot analysis group and a histological examination group. No mice died during the experiment, and each group contained 9 mice.

3. Preparation of drug-treated PISE mice

Eight-three ICR mice with implanted stainless steel tubes were injected (i.p.) with methylscopolamine and pilocarpine, 63 of which displayed category 4–5 seizures. PISE mice were divided into three groups and injected (i.c.v.) with vehicle (vehicle-treated PISE mice), HC-067047 (HC-067047-treated PISE mice), or Ac-YVAD-cmk (Ac-YVAD-cmk-treated PISE mice). Vehicle- and HC-067047-treated PISE mice were randomly divided into a western blot analysis group and a histological examination group. No PISE mice died during the experiment, and each group contained 9 mice.

Eighty-six mice were injected with methylscopolamine and pilocarpine, 63 of which displayed category 4–5 seizures. The PISE mice were randomly divided into three groups and injected (i.p.) with vehicle (vehicle-treated PISE mice), MDL-28170 (MDL-28170-treated PISE mice), or MCC950 (MCC950-treated PISE mice). Mice in each group were randomly divided into a western blot analysis group and a histological examination group. No PISE mice died during the experiment, and each PISE group contained 9 mice.

Supplementary Table 1

Experimental groups

Groups	Drug administration
control-1	ip. injection of methylscopolamine and ip. injection of saline
PISE mice	ip. injection of methylscopolamine and ip. injection of pilocarpine
control-2	ip. injection of vehicle
MDL-28170-injected mice	ip. injection of MDL-28170
MCC950-injected mice	ip. injection of MCC950
Control-3	icv. injection of vehicle
Ac-YVAD-cmk-injected mice	icv. injection of Ac-YVAD-cmk
HC-067047-injected mice	icv. injection of HC-067047
GSK1016790A-injected mice	icv. injection of GSK1016790A
vehicle-injected PISE mice-1 (PISE+vehicle)	ip. injection of methylscopolamine, ip. injection of pilocarpine, and ip. injection of vehicle
MDL-28170-injected PISE mice (PISE+MDL-28170)	ip. injection of methylscopolamine, ip. injection of pilocarpine, and ip. injection of MDL-28170
MCC950-injected PISE mice (PISE+MCC950)	ip. injection of methylscopolamine, ip. injection of pilocarpine, and ip. injection of MCC950
vehicle-injected PISE mice-2 (PISE+vehicle)	ip. injection of methylscopolamine, ip. injection of pilocarpine, and icv. injection of vehicle
Ac-YVAD-cmk-injected PISE mice (PISE+Ac-YVAD-cmk)	ip. injection of methylscopolamine, ip. injection of pilocarpine, and icv. injection of Ac-YVAD-cmk
HC-067047-injected PISE mice (PISE+HC-067047)	ip. injection of methylscopolamine, ip. injection of pilocarpine, and icv. injection of HC-067047

vehicle-injected GSK1016790A-mice-1 (GSK1016790A+vehicle)	icv. injection of GSK1016790A and ip. injection of vehicle
MDL-28170-injected GSK1016790A-mice (GSK1016790A+MDL-28170)	icv. injection of GSK1016790A and ip. injection of MDL-28170
MCC950-injected GSK1016790A-mice (GSK1016790A+MCC950)	icv. injection of GSK1016790A and ip. injection of MCC950
vehicle-injected GSK1016790A-mice-2 (GSK1016790A+vehicle)	icv. injection of GSK1016790A and icv. injection of vehicle
Ac-YVAD-cmk-injected GSK1016790A-mice (GSK1016790A+Ac-YVAD-cmk)	icv. injection of GSK1016790A and icv. injection of Ac-YVAD-cmk

vehicle: saline with 0.1% DMSO

Supplementary Table 2

Tests for normality and variance homogeneity

Figure	experimental group	n	Mean ± SD.	Shapiro-Wilk test		Levene's Test		Median	Interquartile ranges	independent samples *t*-tests		Mann-Whitney U tests
				Statistics	*P* value	*F* value	*P* value		25%~75%	*t* value	*P* value	*P* value
**1A-calpain 1**	control	9	100.00±4.88	0.98	0.98	0.82	0.38			9.42	<0.01	
	PISE	9	74.16±6.63	0.95	0.67							
**1A-calpain 2**	control	9	100.00±6.63	0.90	0.23	2.83	0.11			-0.16	0.87	
	PISE	9	100.44±4.87	0.96	0.75							
**1B-NLRP3**	control	9		0.95	0.63	12.08	<0.01	99.57	96.50~102.93			<0.01
	PISE	9		0.96	0.77			139.95	128.15~153.88			
**1B-c-cas-1**	control	9		0.86	0.10	5.98	0.03	102.06	97.95~103.03			<0.01
	PISE	9		0.90	0.23			135.67	127.04~140.43			
**1B-IL-1β**	control	9		0.91	0.30	9.62	0.01	99.15	96.33~103.50			<0.01
	PISE	9		0.91	0.31			134.44	130.58~149.98			
**1B-N-GSDMD**	control	9		0.97	0.86	11.29	<0.01	98.80	96.72~103.68			<0.01
	PISE	9		0.95	0.65			136.89	130.78~150.68			
**1C-GSDMD^{+} cells**	control	9	4440.00±634.80	0.97	0.85	4.24	0.06			-18.01	<0.01	
	PISE	9	15178.00±1672.40	0.93	0.53							
**2A- calpain 1**	PISE+vehicle	9		0.99	0.99	4.98	0.04	101.65	97.19~103.72			<0.01
	PISE+MDL	9		0.93	0.50			146.18	134.92~151.96			
**2B- NLRP3**	PISE+vehicle	9	100.00±6.26	0.89	0.20	0.01	0.91			0.74	0.47	
	PISE+MDL	9	97.78±6.43	0.89	0.21							
**2B- c-cas-1**	PISE+vehicle	9	100.00±5.93	0.94	0.56	3.07	0.10			7.81	<0.01	
	PISE+MDL	9	68.08±10.73	1.00	1.00							
**2B- IL-1β**	PISE+vehicle	9	100.00±5.73	0.79	0.06	4.22	0.06			6.30	<0.01	
	PISE+MDL	9	72.13±11.98	0.84	0.06							
**2B- N-GSDMD**	PISE+vehicle	9		0.93	0.48	9.17	0.01	98.36	96.46~100.67			<0.01

		PISE+MDL	9		0.98	0.97			72.64	63.87~78.48			
**2C- GSDMD^{+} cells**		PISE+vehicle	9	15380.00±1889.75	0.97	0.91	0.42	0.53			8.83	<0.01	
		PISE+MDL	9	8240.00±1517.82	0.89	0.19							
**2E-i**	**CA1**	control	9	100.00±4.94	0.92	0.39	3.82	0.07			22.76	<0.01	
		PISE	9	57.52±2.62	0.94	0.58							
	**CA2/3**	control	9	100.00±3.88	0.86	0.10	3.04	0.10			28.02	<0.01	
		PISE	9	57.37±2.40	0.90	0.24							
**2E-ii**	**CA1**	PISE+vehicle	9	100.00±7.37	0.93	0.53	1.01	0.98			6.88	<0.01	
		PISE+MDL	9	135.85±7.32	0.91	0.31							
	**CA2/3**	PISE+vehicle	9	100.00±7.65	0.92	0.46	1.58	0.52			6.15	<0.01	
		PISE+MDL	9	136.29±9.64	0.91	0.33							
**3A- c-cas-1**		control	9		0.95	0.73	7.35	0.02	98.74	97.50~102.58			<0.01
		MCC950	9		0.90	0.28			94.10	86.11~96.81			
**3A- IL-1β**		control	9	100.56±3.66	0.93	0.47	0.39	0.54			5.75	<0.01	
		MCC950	9	89.95±4.15	0.96	0.83							
**3A- N-GSDMD**		control	9	100.00±3.45	0.94	0.61	0.65	0.43			5.98	<0.01	
		MCC950	9	88.56±4.60	0.97	0.92							
**3B- c-cas-1**		control	9	100.00±4.61	0.89	0.21	0.01	0.93			5.04	<0.01	
		Ac	9	89.04±4.62	0.91	0.31							
**3B- IL-1β**		control	9	100.00±2.97	0.92	0.42	0.67	0.42			5.91	<0.01	
		Ac	9	90.71±3.67	0.95	0.73							
**3B- N-GSDMD**		control	9	100.00±3.74	0.90	0.27	1.07	0.32			8.14	<0.01	
		Ac	9	88.10±2.28	0.98	0.98							
**3C - c-cas-1**		PISE+vehicle	9	100.00±3.05	0.90	0.27	3.46	0.08			10.47	<0.01	
		PISE+MCC	9	74.67±6.59	0.96	0.75							
**3C - IL-1β**		PISE+vehicle	9	100.00±7.79	0.86	0.09	0.33	0.57			8.50	<0.01	
		PISE+MCC	9	71.67±6.27	0.97	0.92							
**3C-N-GSDMD**		PISE+vehicle	9		0.96	0.84	7.00	0.02	99.77	97.42~102.08			<0.01
		PISE+MCC	9		0.87	0.12			78.13	69.99~82.96			
**3D c-cas-1**		PISE+vehicle	9	100.00±3.94	0.92	0.36	4.17	0.06			9.50	<0.01	
		PISE+Ac	9	73.22±7.48	0.89	0.20							

**3D - IL-1β**		PISE+vehicle	9	100.00±3.42	0.94	0.54	0.82	0.38			11.54	<0.01	
		PISE+Ac	9	76.87±4.95	0.85	0.08							
**3D - N-GSDMD**		PISE+vehicle	9	100.00±3.66	0.94	0.63	1.49	0.24			12.08	<0.01	
		PISE+Ac	9	73.59±5.45	0.94	0.60							
**3E**	**CA1**	PISE+vehicle	9	100.00±7.37	0.93	0.53	1.55	0.54			10.01	<0.01	
		PISE+MCC	9	144.55±5.91	0.92	0.46							
	**CA2/3**	PISE+vehicle	9	100.00±7.65	0.92	0.46	2.98	0.14			14.50	<0.01	
		PISE+MCC	9	155.80±4.43	0.93	0.51							
**4A-calpain1**		control	9		0.92	0.42	7.32	0.02	102.01	95.08~104.86			0.01
		HC-067047	9		0.89	0.19			112.71	99.75~118.97			
**4B-NLRP3**		control	9	100.00±3.30	0.90	0.24	0.23	0.64			6.49	<0.01	
		HC-067047	9	90.99±2.54	0.98	0.97							
**4B-c-cas-1**		control	9	100.00±3.43	0.87	0.13	0.85	0.37			6.53	<0.01	
		HC-067047	9	90.44±2.73	0.98	0.94							
**4B-IL-1β**		control	9	100.00±3.44	0.97	0.91	0.16	0.69			4.23	<0.01	
		HC-067047	9	93.04±3.55	0.95	0.70							
**4B-N-GSDMD**		control	9	100.00±4.15	0.91	0.33	3.45	0.08			5.28	<0.01	
		HC-067047	9	91.36±2.62	0.96	0.81							
**4C-calpain1**		PISE+vehicle	9	100.00±3.80	0.94	0.60	2.44	0.14			-17.09	<0.01	
		PISE+HC	9	140.94±6.10	0.96	0.84							
**4D-NLRP3**		PISE+vehicle	9		0.93	0.53	4.77	0.04	99.77	96.67~102.85			<0.01
		PISE+HC	9		0.94	0.58			74.11	66.62~80.53			
**4D-c-cas-1**		PISE+vehicle	9	100.00±3.41	0.90	0.24	2.98	0.10			9.38	<0.01	
		PISE+HC	9	75.64±7.01	0.97	0.87							
**4D-IL-1β**		PISE+vehicle	9	100.11±3.92	0.92	0.43	0.37	0.55			12.31	<0.01	
		PISE+HC	9	74.63±4.82	0.96	0.84							
**4D-N-GSDMD**		PISE+vehicle	9		0.93	0.50	12.03	<0.01	99.10	98.11~102.00			<0.01
		PISE+HC	9		0.92	0.37			73.42	66.29~85.20			
**4E-i-GSDMD⁺ cells**		control	9	4390.00 ±219.10	0.92	0.40	4.01	0.06			4.34	<0.01	
		HC-067047	9	4020.00±135.10	0.98	0.96							

**4F-i-GSDMD⁺ cells**		PISE+vehicle	9		0.91	0.31	22.55	<0.01	14000.00	12815.00~16970.00			<0.01
		PISE+HC	9		0.89	0.19			8270.00	7835.00~8500.00			
**G-i**	**CA1**	control	9	100.00±4.17	0.98	0.97	1.13	0.86			5.14	<0.01	
		HC-067047	9	109.83±3.92	0.92	0.44							
	**CA2/3**	control	9	100.00±4.81	0.90	0.28	2.60	0.19			2.35	0.03	
		HC-067047	9	107.16±7.77	0.90	0.26							
**G-ii**	**CA1**	PISE+vehicle	9	100.00±7.12	0.95	0.76	1.45	0.6			10.12	<0.01	
		PISE+HC	9	148.23±5.90	0.92	0.46							
	**CA2/3**	PISE+vehicle	9	100.00±5.83	0.92	0.43	1.10	0.88			14.20	<0.001	
		PISE+HC	9	151.13±5.54	0.94	0.67							
**5A-calpain 1**		control	9	100.00±3.53	0.87	0.13	0.58	0.46			12.72	<0.01	
		GSK	9	73.69±5.10	0.99	1.00							
**5A-calpain 2**		control	9	100.00±4.29	0.91	0.28	0.01	0.96			0.19	0.86	
		GSK	9	99.65±3.76	0.97	0.91							
**5B-NLRP3**		control	9		0.95	0.71	16.25	<0.01	99.88	97.87~101.56			<0.01
		GSK	9		0.93	0.51			134.90	127.42~149.43			
**5B-c-cas-1**		control	9		0.89	0.18	5.50	0.03	98.03	97.38~102.72			<0.01
		GSK	9		0.79	0.02			144.82	133.13~152.48			
**5B-IL-1β**		control	9		0.83	0.04	11.43	<0.01	98.21	97.56~103.03			<0.01
		GSK	9		0.91	0.35			146.26	131.41~151.59			
**5B-N-GSDMD**		control	9		0.93	0.48	6.14	0.02	101.50	95.32~103.10			<0.01
		GSK	9		0.99	0.99			145.72	134.91~157.29			
**5C-GSDMD⁺ cells**		control	9	4230.00±418.50	0.89	0.22	0.62	0.44			-20.96	<0.01	
		GSK	9	9160.00±568.40	0.94	0.53							
**5E-calpain1**		GSK+vehicle	9		0.98	0.95	19.01	<0.01	99.82	97.50~102.83			<0.01
		GSK+MDL	9		0.93	0.44			138.71	123.22~154.32			
**5F-NLRP3**		GSK+vehicle	9	100.00±2.89	0.97	0.89	2.84	0.11			-0.20	0.85	
		GSK+MDL	9	99.32±3.94	0.90	0.27							
**5F-c-cas-1**		GSK+vehicle	9	100.00±4.42	0.91	0.29	1.47	0.24			8.73	<0.01	
		GSK+MDL	9	73.57±7.93	0.96	0.81							
**5F-IL-1β**		GSK+vehicle	9	100.00±3.78	0.95	0.68	1.46	0.24			9.35	<0.01	

	GSK+MDL	9	75.06±7.05	0.95	0.69							
**5F-N-GSDMD**	GSK+vehicle	9	100.00±3.21	0.93	0.50	2.40	0.14			13.52	<0.01	
	GSK+MDL	9	71.89±5.35	0.93	0.51							
**5G-c-cas-1**	GSK+vehicle	9	100.00±3.88	0.93	0.51	3.17	0.09			8.17	<0.01	
	GSK+MCC	9	74.36±8.57	0.99	0.99							
**5G-IL-1β**	GSK+vehicle	9	100.00±3.27	0.93	0.48	3.02	0.10			11.62	<0.01	
	GSK+MCC	9	74.63±5.68	0.92	0.38							
**5G-N-GSDMD**	GSK+vehicle	9		0.90	0.28	3.99	0.06	99.57	97.65~102.58			<0.01
	GSK+MCC	9		0.80	0.02			69.20	64.27~75.62			
**5H-c-cas-1**	GSK+vehicle	9		0.89	0.20	30.36	<0.01	99.94	98.29~100.67			<0.01
	GSK+Ac	9		0.87	0.13			65.00	57.92~77.32			
**5H-IL-1β**	GSK+vehicle	9		0.97	0.89	7.76	0.01	100.30	97.34~102.23			<0.01
	GSK+Ac	9		0.96	0.76			70.04	61.88~78.16			
**5H-N-GSDMD**	GSK+vehicle	9	100.00±3.38	0.97	0.90	2.81	0.11			13.38	<0.01	
	GSK+Ac	9	69.62±5.91	0.91	0.35							

calpain 1: inactivated/total calpain 1 ratio, calpain 2: inactivated/total calpain 2 ratio, MDL: MDL-28170, MCC: MCC950, Ac: Ac-YVAD-cmk, HC: HC-067047, GSK: GSK1016790A

Supplementary Table 3

Tests for normality and variance homogeneity

	experimental group	n	Mean ± SD.	Shapiro-Wilk test		Levene's Test		Median	Interquartile ranges	independent samples *t*-tests		Mann-Whitney U tests
				Statistics	*P* value	*F* value	*P* value		25%~75%	*t* value	*P* value	*P* value
**calpain 1**	control	9	100.00±4.61	0.91	0.28	0.84	0.37			1.07	0.30	
	MDL-28170	9	97.84±.392	0.92	0.40							
**NLRP3**	control	9	100.00±6.05	0.91	0.29	1.51	0.29			1.02	0.33	
	MDL-28170	9	96.59±8.07	0.87	0.13							
**c-cas-1**	control	9	100.00±4.96	0.97	0.86	2.04	0.17			1.17	0.26	
	MDL-28170	9	97.58±3.76	0.97	0.86							
**IL-1β**	control	9	100.00±7.30	0.87	0.13	0.44	0.84			0.89	0.39	
	MDL-28170	9	97.05±6.86	0.86	0.09							
**N-GSDMD**	control	9	100.00±5.98	0.95	0.64	0.001	0.98			0.37	0.72	
	MDL-28170	9	99.62±5.10	0.91	0.29							
**GSDMD$^+$ cells**	control	9	4396.67±250.75	0.94	0.59	2.36	0.14			0.47	0.65	
	MDL-28170	9	4391.11±547.44	0.97	0.89							

calpain 1: inactivated/total calpain 1

Supplementary Table 4

Tests for normality and variance homogeneity

	experimental group	n	Mean ± SD.	Shapiro-Wilk test		Levene's Test		Median	Interquartile ranges	independent samples *t*-tests		Mann-Whitney U tests
				Statistics	*P* value	*F* value	*P* value		25%~75%	*t* value	*P* value	*P* value
**calpain 1**	control	9		0.83	0.06	8.12	0.01	96.2931	91.64~110.40			0.35
	HC-067047	9		0.93	0.47			95.0365	91.42~99.14			
**NLRP3**	control	9	100.00±6.05	0.91	0.29	1.51	0.24			1.02	0.33	
	HC-067047	9	96.59±8.07	0.87	0.18							
**c-cas-1**	control	9		0.97	0.860	4.83	0.04	97.5588	94.52~106.63			0.42
	HC-067047	9		0.97	0.86			97.4068	93.47~100.11			
**IL-1β**	control	9	100.00±7.30	0.87	0.13	0.04	0.84			0.89	0.39	
	HC-067047	9	97.05±6.86	0.86	0.10							
**N-GSDMD**	control	9	100.66±5.98	0.94	0.64	0.001	0.98			0.37	0.72	
	HC-067047	9	101.62±5.09	0.91	0.29							
**GSDMD^{+} cells**	control	9		0.94	0.59	5.20	0.04	4340.0000	4155.00~4625.00			0.77
	HC-067047	9		0.97	0.89			4580.0000	3780.00~4765.00			

calpain 1: inactivated/total calpain 1

Supplementary Figure 1

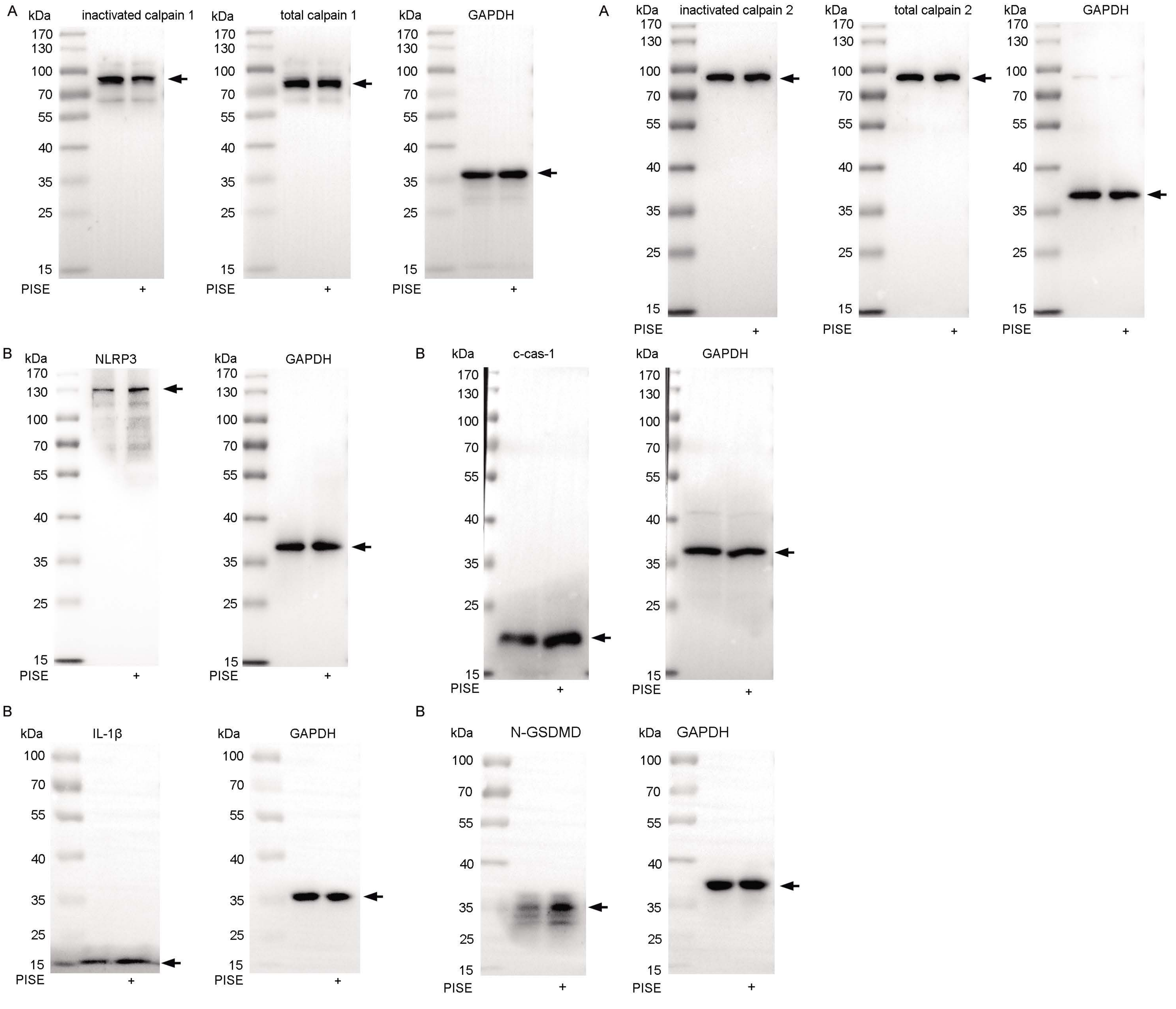

Supplementary Figure 2

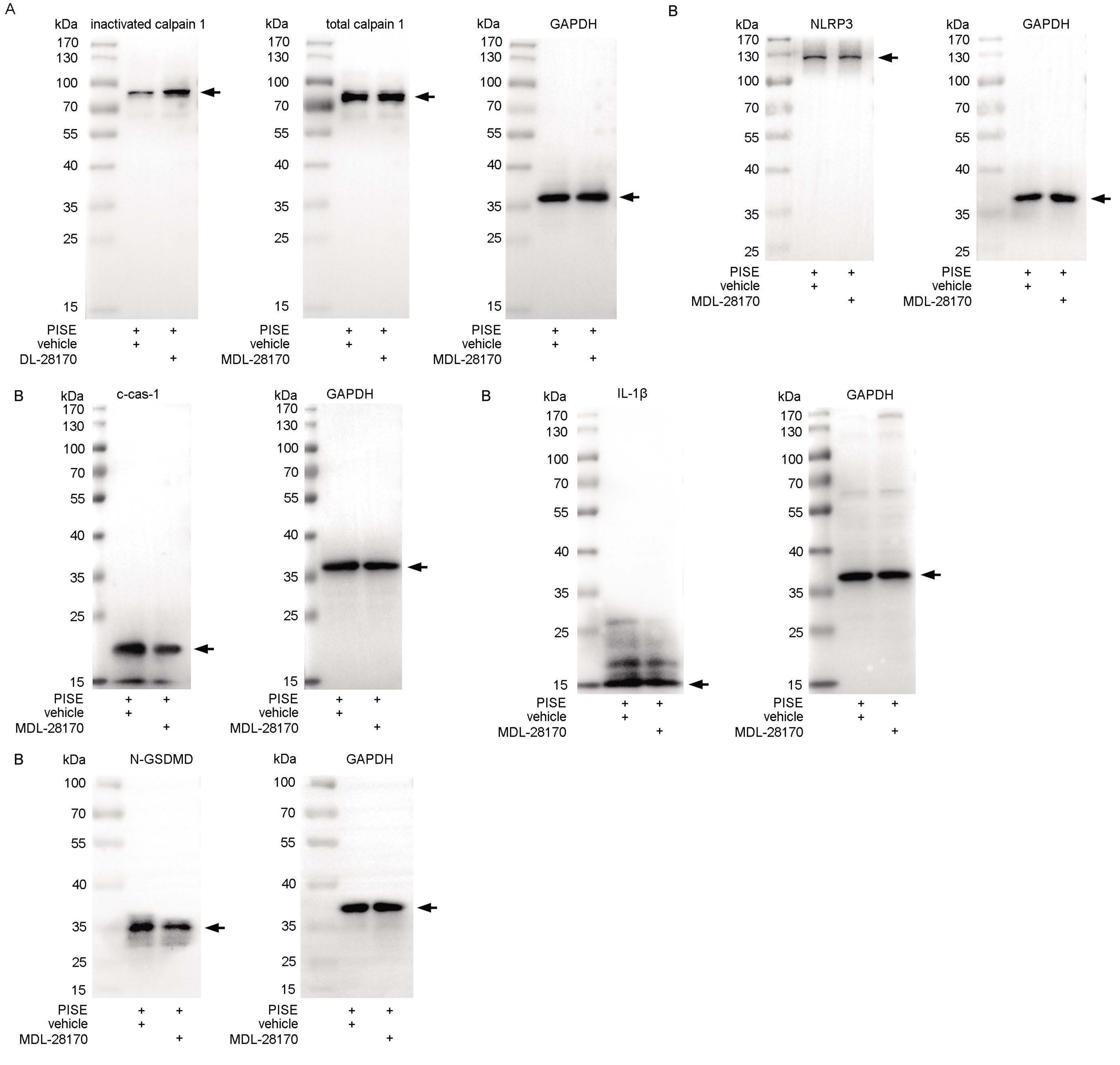

Supplementary Figure 3

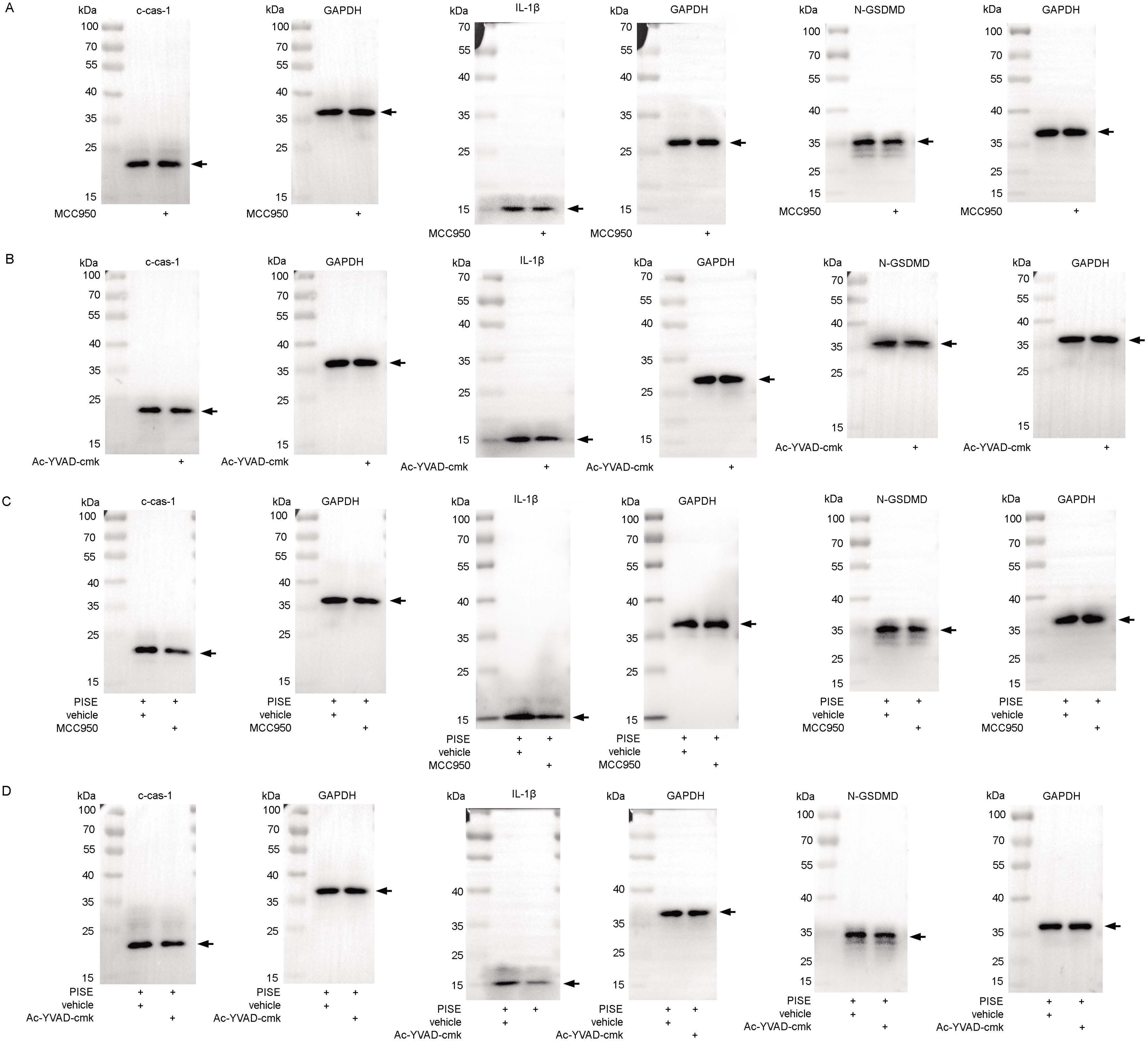

Supplementary Figure 4

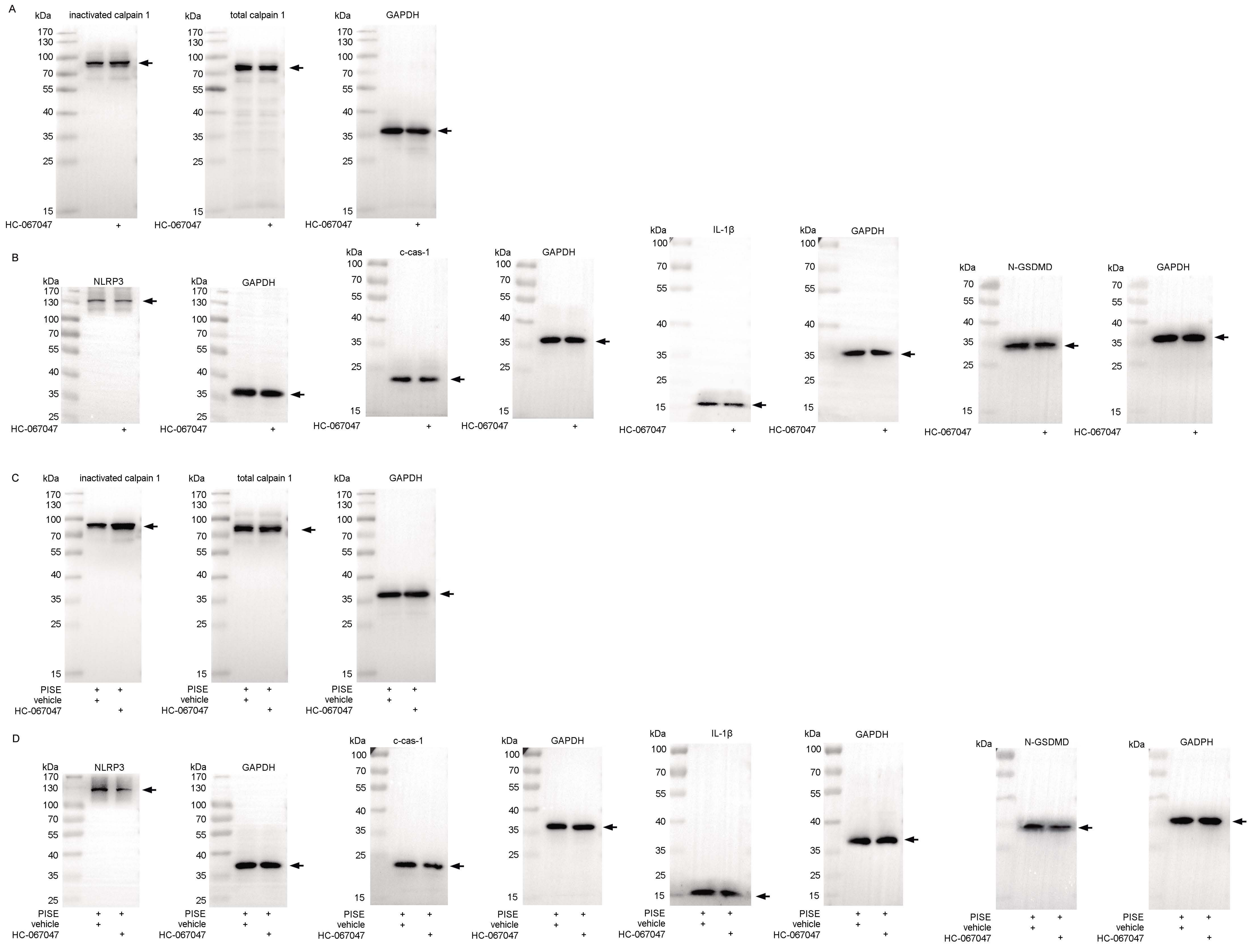

Supplementary Figure 5

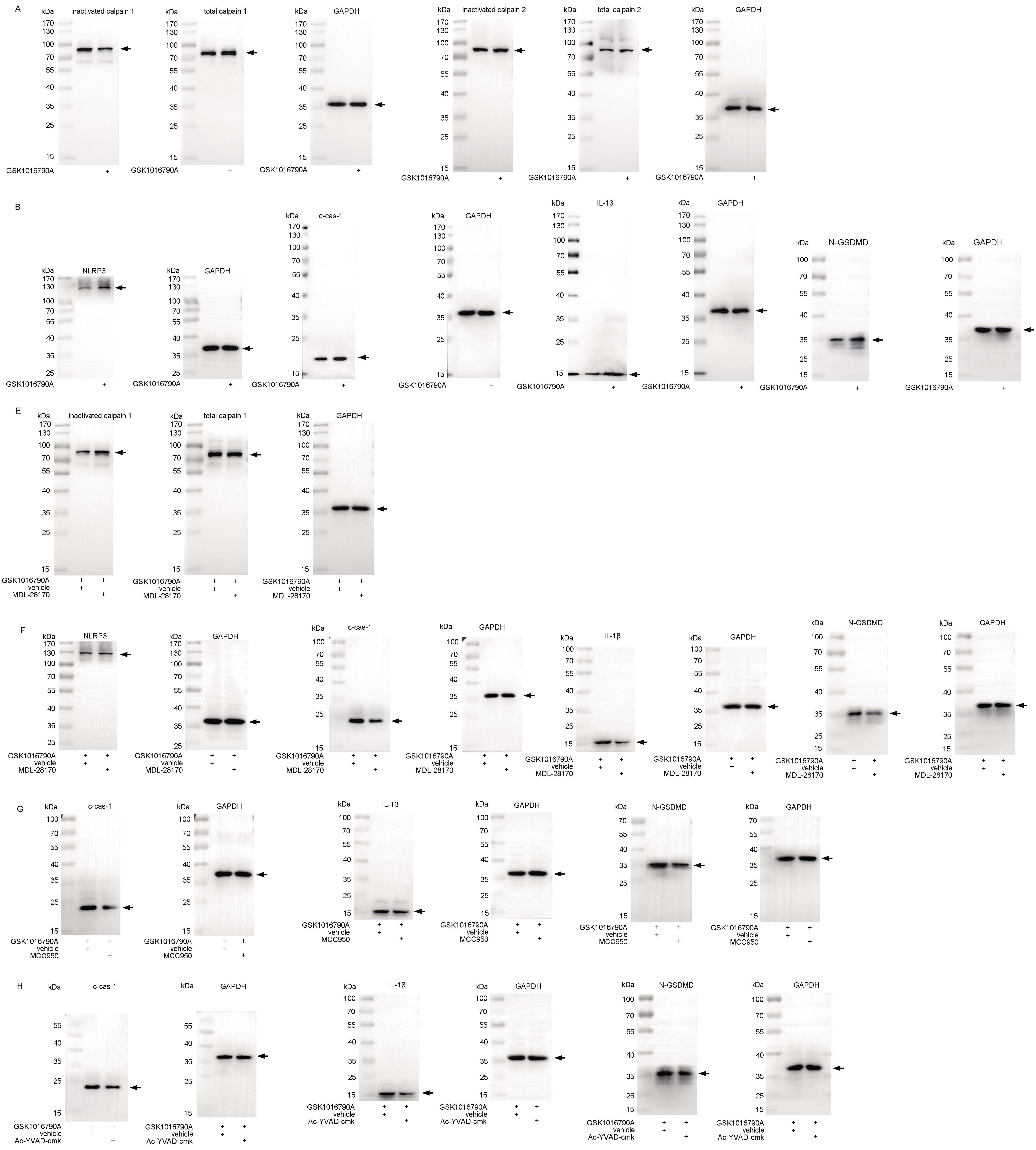

Supplement: Supplementary file 1 — Supplementary Material 1 [file 40478_2025_1990_MOESM1_ESM.pdf]
